# Supplementary material for: Hierarchical Li electrochemistry using alloy-type anode for high-energy-density Li metal batteries
Source: Nat Commun. 2024 Feb 14;15:1354. doi: 10.1038/s41467-024-45613-4 (PMC10867008; doi:10.1038/s41467-024-45613-4)
Supplement: Supplementary file 1 — Supplementary Information [file 41467_2024_45613_MOESM1_ESM.pdf]

Hierarchical Li electrochemistry using alloy-type anode for high-energy-density Li metal batteries

Jiaqi Cao[1,#], Yuansheng Shi[1,#], Aosong Gao[2], Guangyuan Du[1], Muhtar Dilxat[1], Yongfei Zhang[1], Mohang Cai[1], Guoyu Qian[1], Xueyi Lu[1], Fangyan Xie[2], Yang Sun[1], and Xia Lu[1,*]

[1]*School of Materials, Sun Yat-sen University, Shenzhen 518107, P.R. China.*

[2]*Instrumental Analysis & Research Center, Sun Yat-sen University, Guangzhou, 510275, PR China.*

[#]Jiaqi Cao and Yuansheng Shi contributed equally to this work.

Email: luxia3@mail.sysu.edu.cn

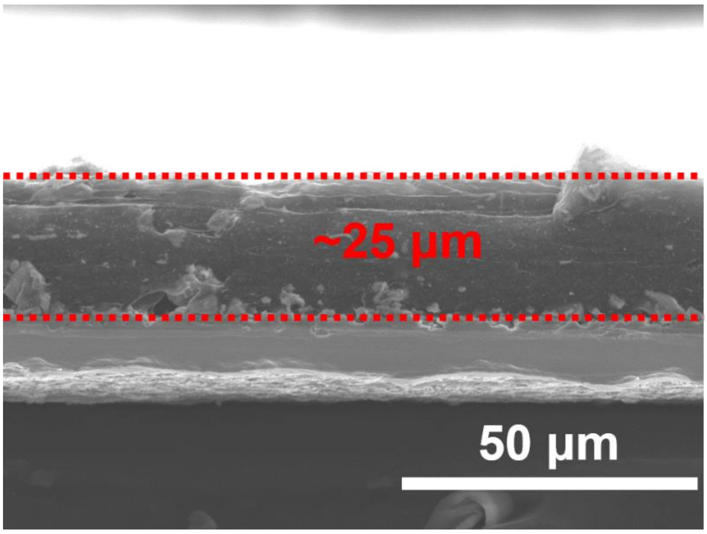

Supplementary Fig. 1. Cross-section SEM images of the fabricated thin Li/LiZn@Cu anode.

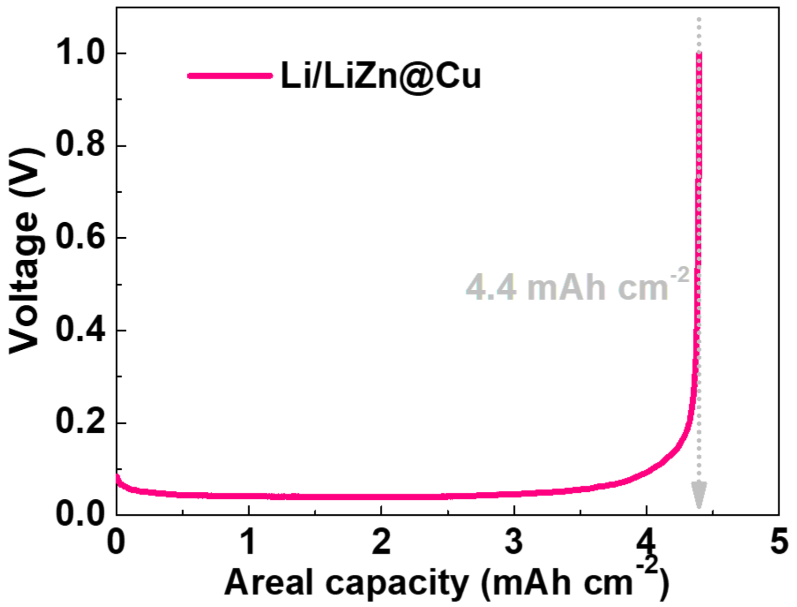

Supplementary Fig. 2. Voltage profile of the stripping process for the thin Li/LiZn@Cu (25 μm-thick Li/LiZn layer).

Supplementary Table 1. Calculated energy densities of the commercial graphite anode.

Parameter	Value
Weight	10 mg cm^{-2}
Thickness	59 μm
Capacity	3.5 mAh cm^{-2}
Volumetric energy density	593.2 mAh cm^{-3}
Gravimetric energy density	350 mAh g^{-1}

The parameters referred to Small 2023, 19, 2205653[1].

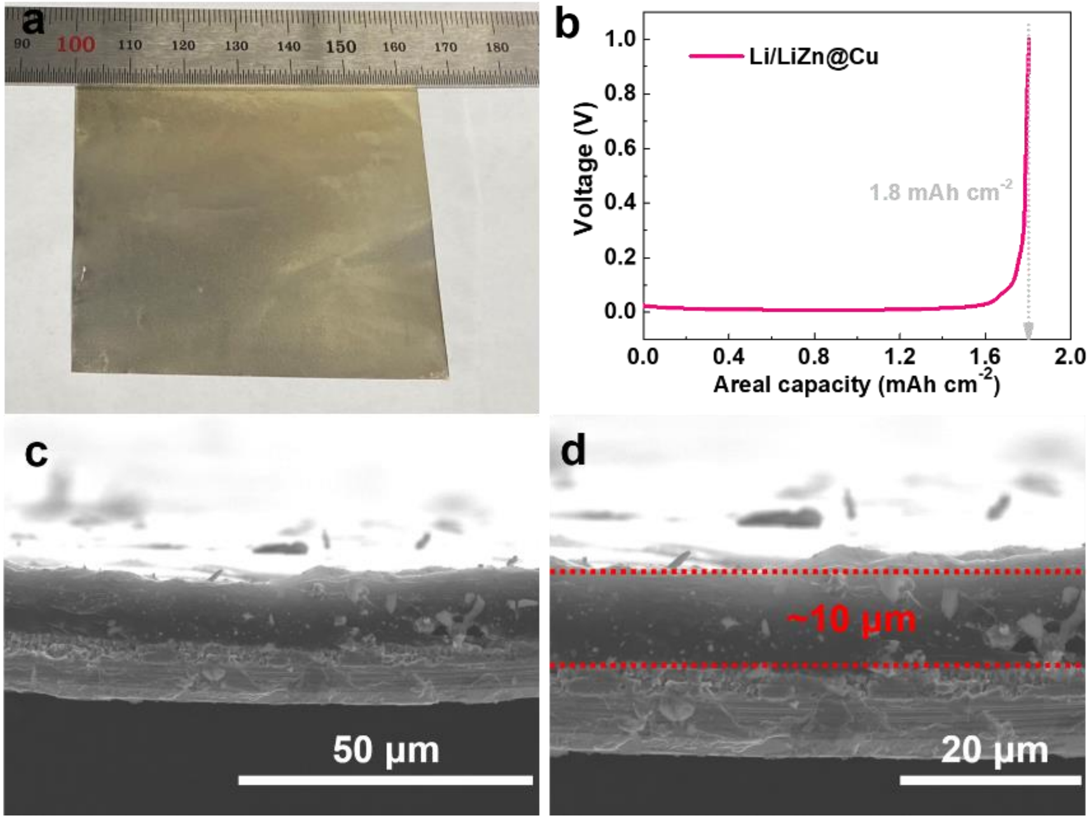

Supplementary Fig. 3. Characteristics of the Li/LiZn@Cu anode with 10 μm-thick Li/LiZn layer. (a) optical photos of the fabricated Li/LiZn@Cu anode. (b) Voltage profile of the stripping process for the thin Li/LiZn@Cu. (c-d) Cross-section SEM images of the thin Li/LiZn@Cu anode.

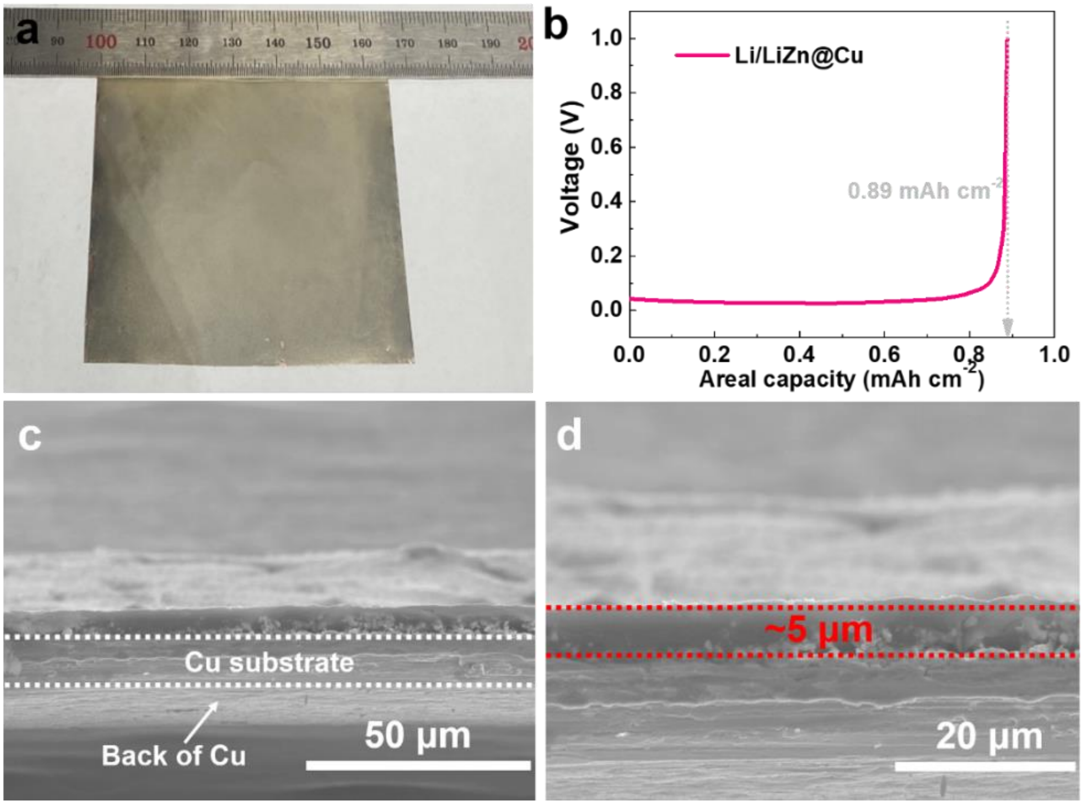

Supplementary Fig. 4. Characteristics of the Li/LiZn@Cu anode with 5 μm-thick Li/LiZn layer. (a) optical photos of the fabricated Li/LiZn@Cu anode. (b) Voltage profile of the stripping process for the thin Li/LiZn@Cu. (c-d) Cross-section SEM images of the thin Li/LiZn@Cu anode.

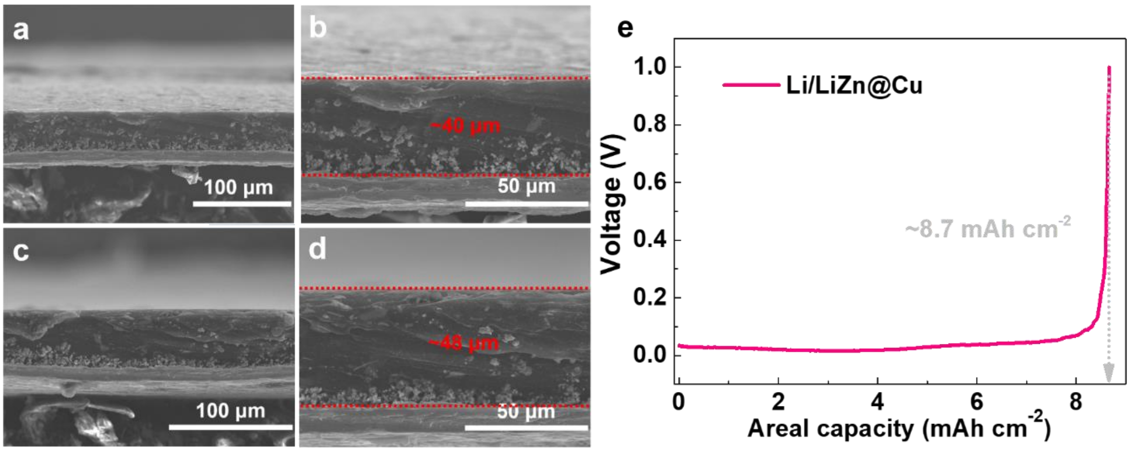

Supplementary Fig. 5. Cross-section SEM images of Li/LiZn@Cu with (a-b) 40 μm-thick and (c-d) 48 μm-thick Li/LiZn layers. (e) Voltage profile of the stripping process for the Li/LiZn@Cu with 48 μm-thick Li/LiZn layer.

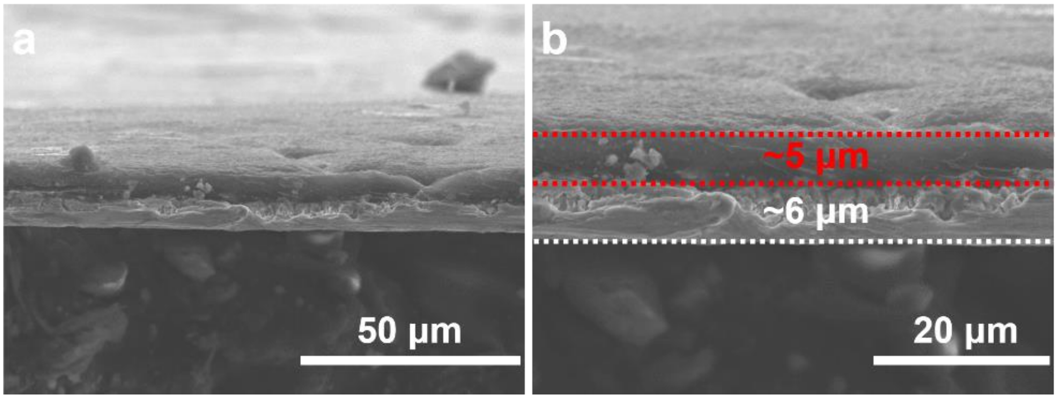

Supplementary Fig. 6. (a-b) Cross-section SEM images of the fabricated thin Li/LiZn@Cu anode (5 μm-thick Li/LiZn) with thin Cu substrate (6 μm).

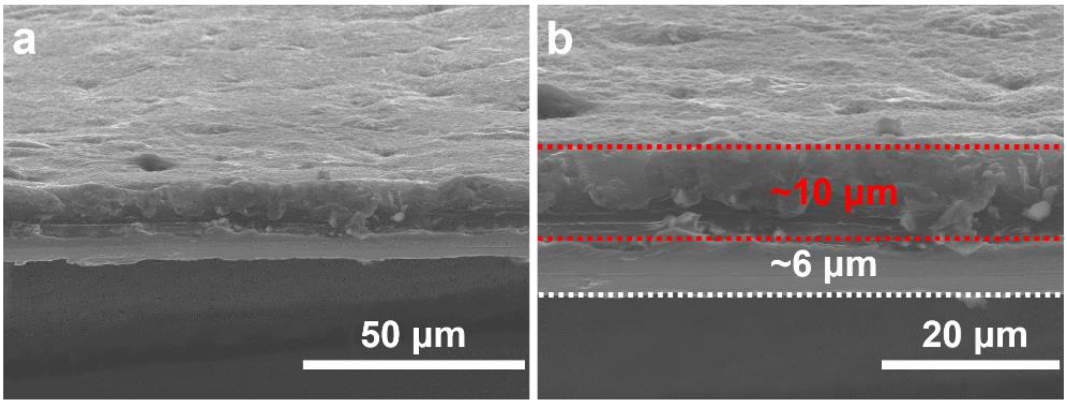

Supplementary Fig. 7. (a-b) Cross-section SEM images of the fabricated thin Li/LiZn@Cu anode (10 μm-thick Li/LiZn) with thin Cu substrate (6 μm).

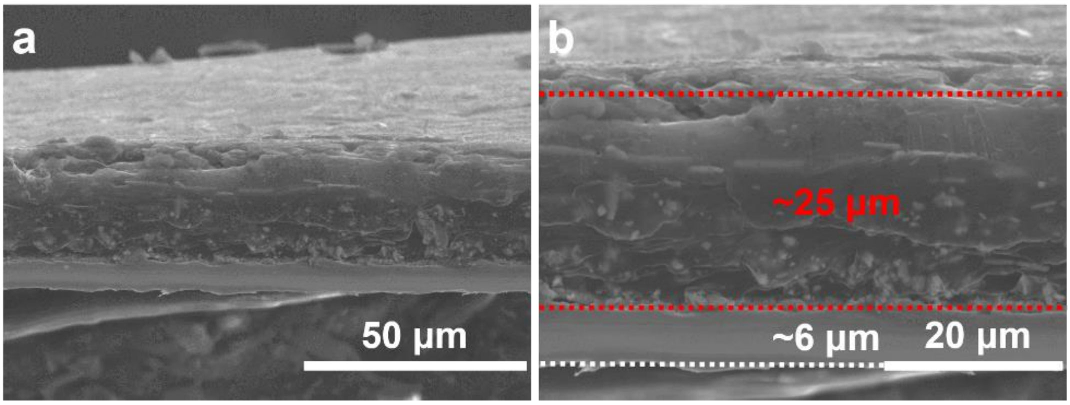

Supplementary Fig. 8. (a-b) Cross-section SEM images of the fabricated thin Li/LiZn@Cu anode (25 μm-thick Li/LiZn) with thin Cu substrate (6 μm).

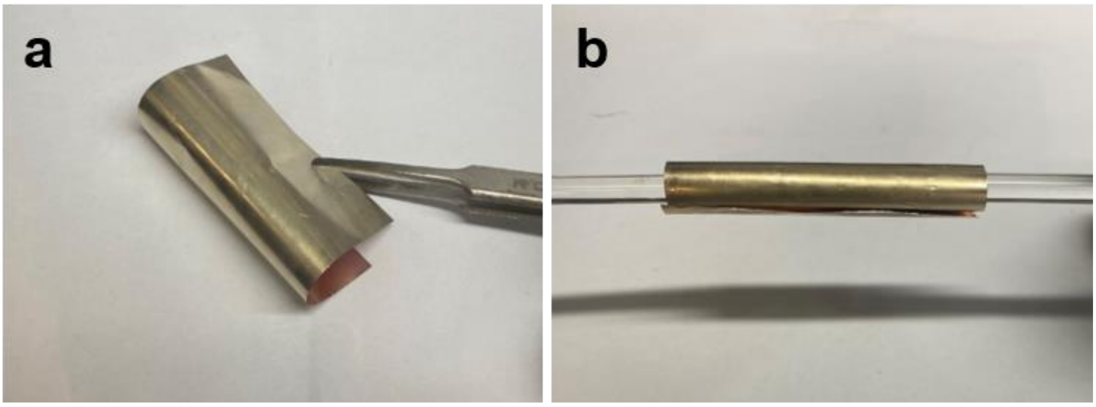

Supplementary Fig. 9. Optic photos of the flexibility of the thin Li/LiZn@Cu anode under (a) bending and (b) coiling states.

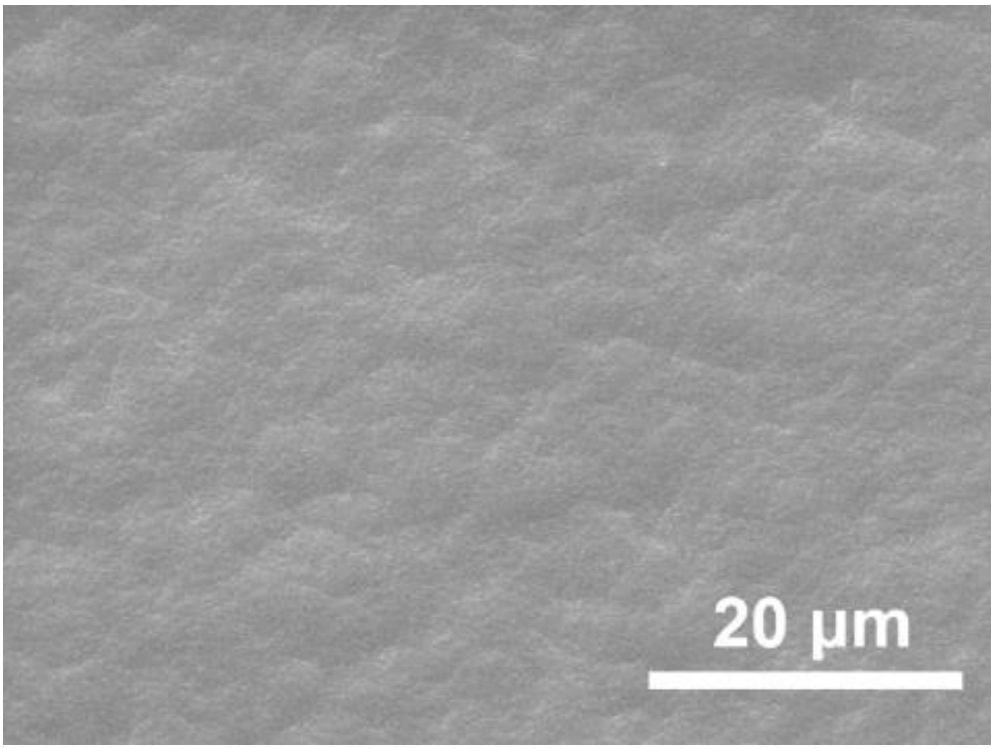

Supplementary Fig. 10. Top-view SEM image of pure Cu foil.

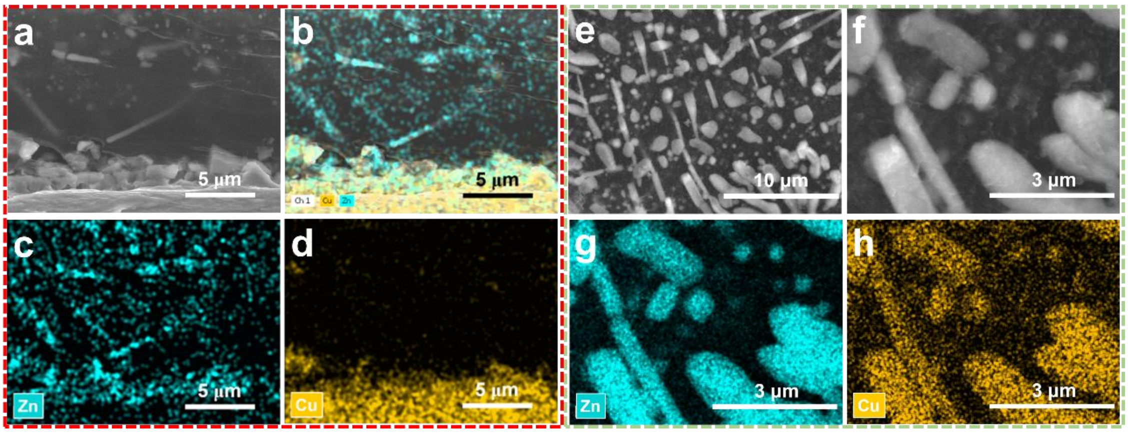

Supplementary Fig. 11. (a) Cross-section SEM image and (b-d) the corresponding EDS mapping of the interface between the Li/LiZn layer and Cu substrate. (e-f) Top-view SEM images and (g-h) the corresponding EDS mapping of the Li/LiZn@Cu after partial Li stripping.

Due to the intermetallic compound LiZn alloy in the molten Li-Zn mixture, the LiZn alloy will be phase-segregated and form a 3D framework together with the Li_2ZnCu_3 alloy, in which the metallic Li fills the surrounding area of the framework after a cooling process. Thus, excepting for the nanodot-like structures, there are obvious nanorod-like (i.e. pillar-like) structures present on the Li/LiZn@Cu surface. It is worth noting that these nanodots correspond to the nanorods buried in the Li layer, resulting in the exposure of the top part only. As shown in Supplementary Fig. 11a-d, some nanorods can be observed inside the Li/LiZn layer. Moreover, due to a higher oxidization potential of LiZn alloy with respect to the metallic Li, the LiZn alloy will maintain its composition and crystal structure before the complete stripping of the surrounding Li ions to act firstly as a framework. Thus, after partial Li stripping, more previously buried alloy-based nanorods can be observed (Supplementary Fig. 11e-h).

Supplementary Table 2. EDS results of the Fig. 1j.

Element	Atom (%)
Zn	89.09
Cu	10.91

Supplementary Table 2 shows the EDS results of Fig. 1j, in which the concentration of Cu element is only 10.91%. According to the chemical formulas of Li_2ZnCu_3 and LiZn, the content of Li_2ZnCu_3 in alloy-based nanostructures is max. 4.1%.

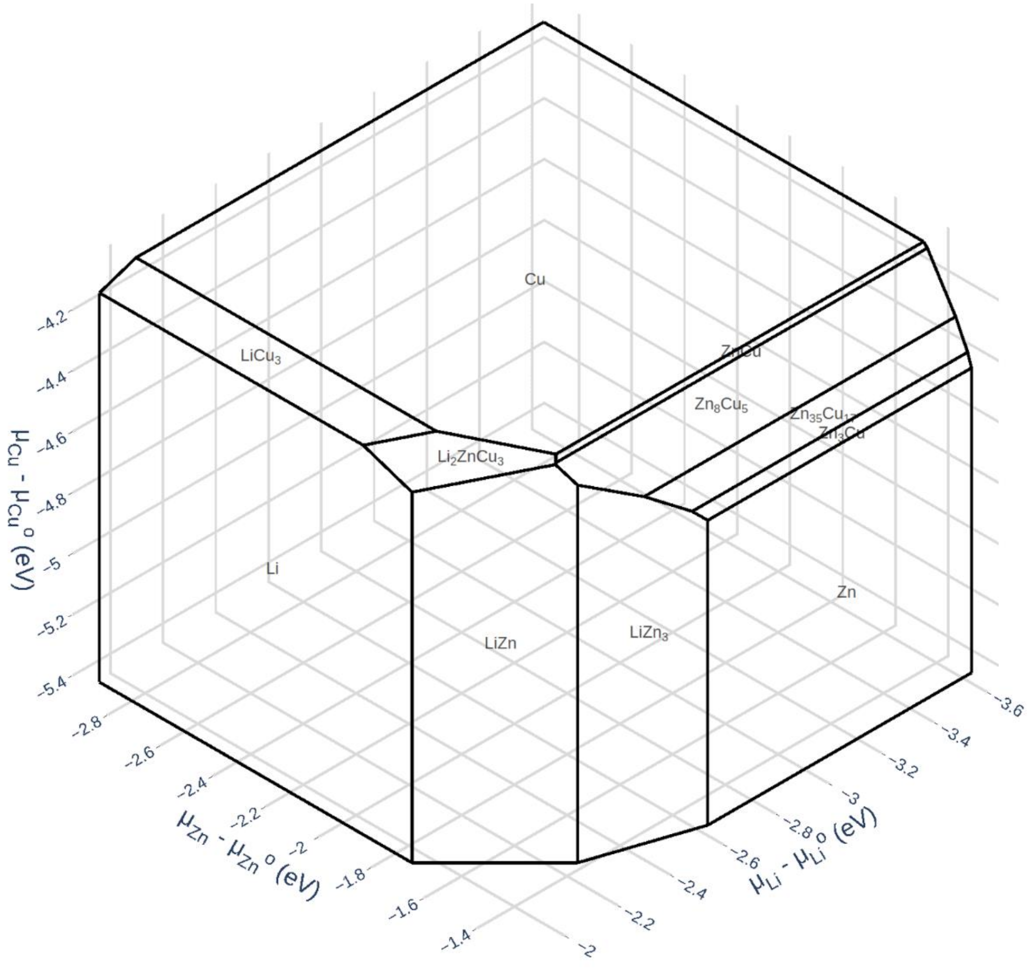

0 K

Supplementary Fig. 12. Chemical potential diagrams of Li-Zn-Cu system at 0 K.

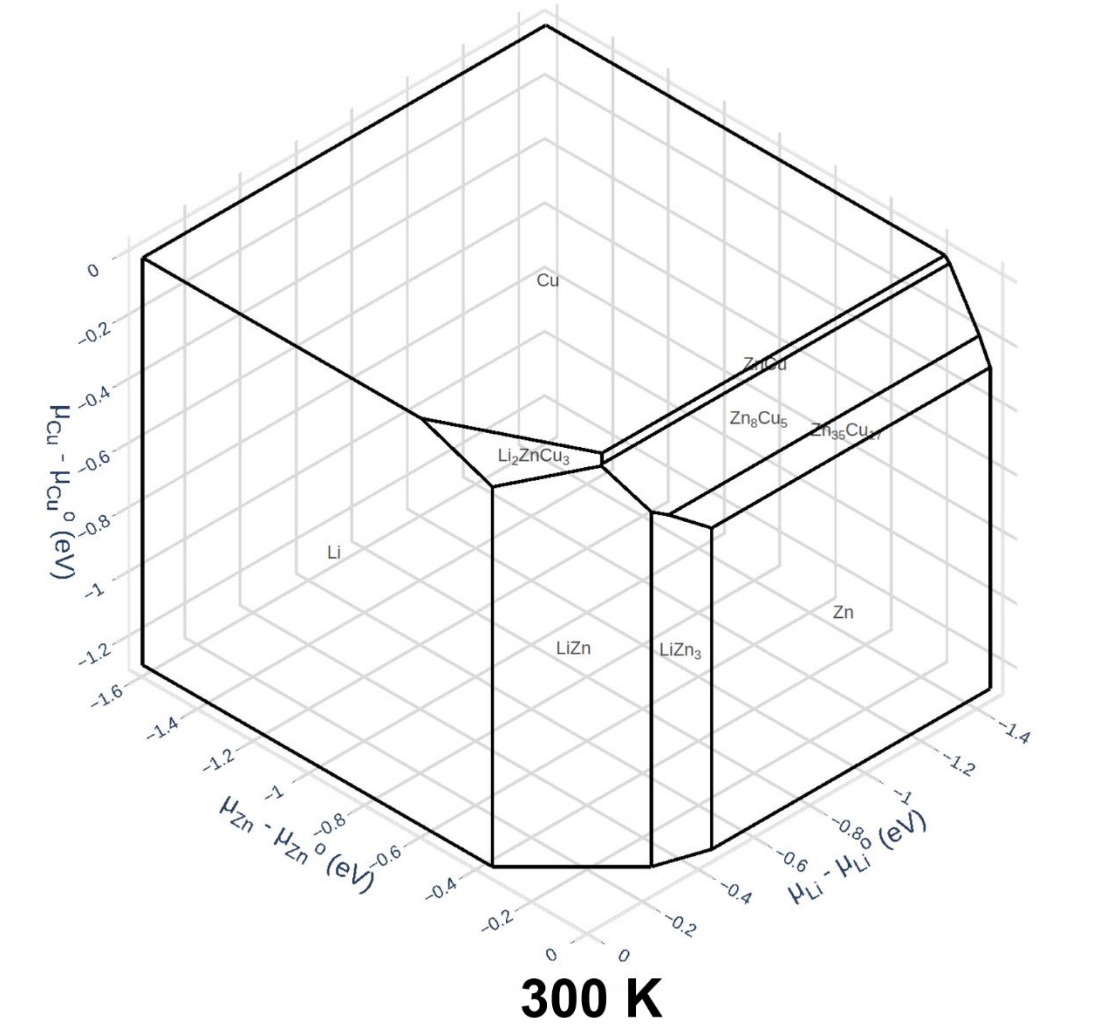

Supplementary Fig. 13. Chemical potential diagrams of Li-Zn-Cu system at 300 K.

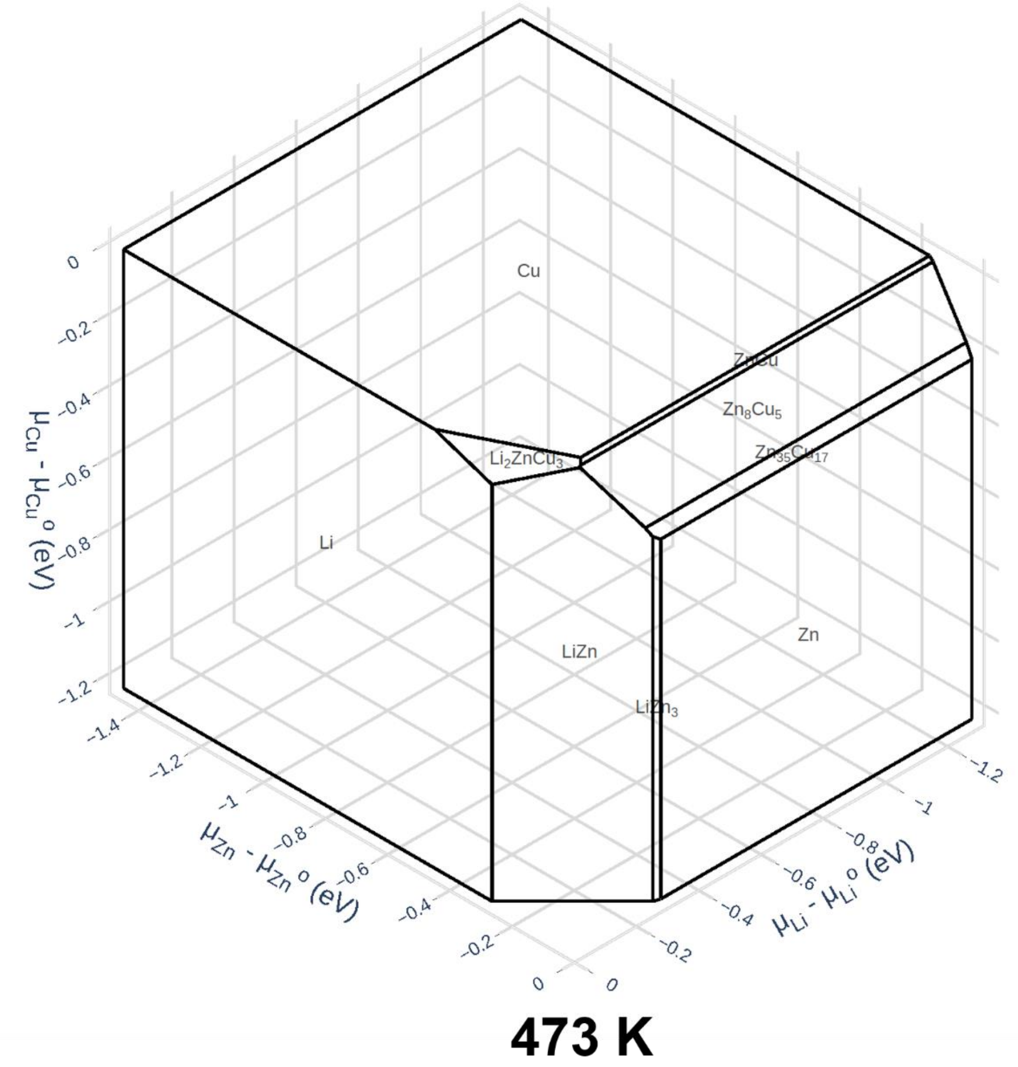

Supplementary Fig. 14. Chemical potential diagrams of Li-Zn-Cu system at 473 K, corresponding to 200 ºC.

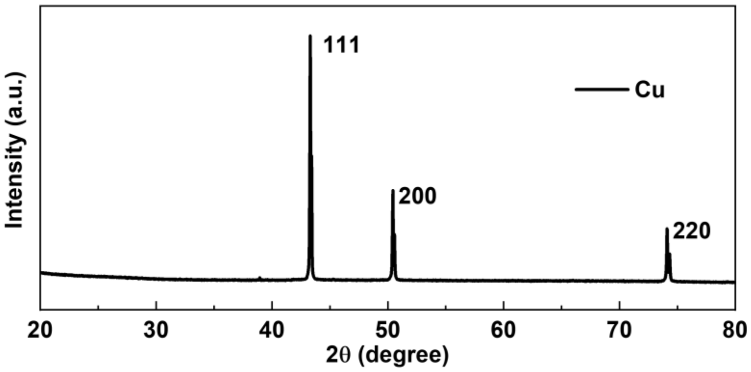

Supplementary Fig. 15. XRD pattern of the pure Cu sample.

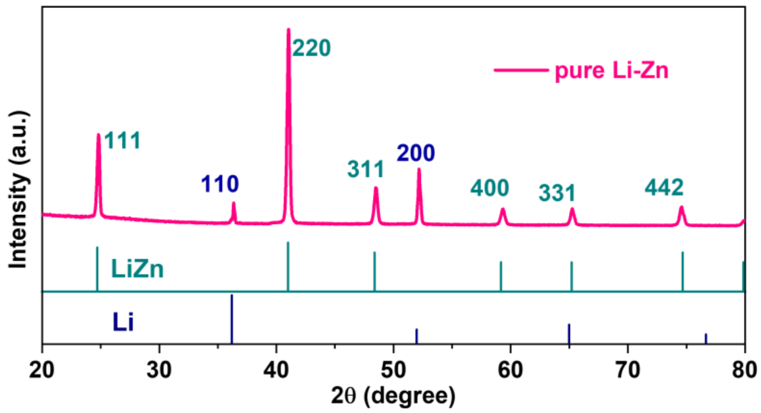

Supplementary Fig. 16. XRD pattern of the pure Li-Zn sample.

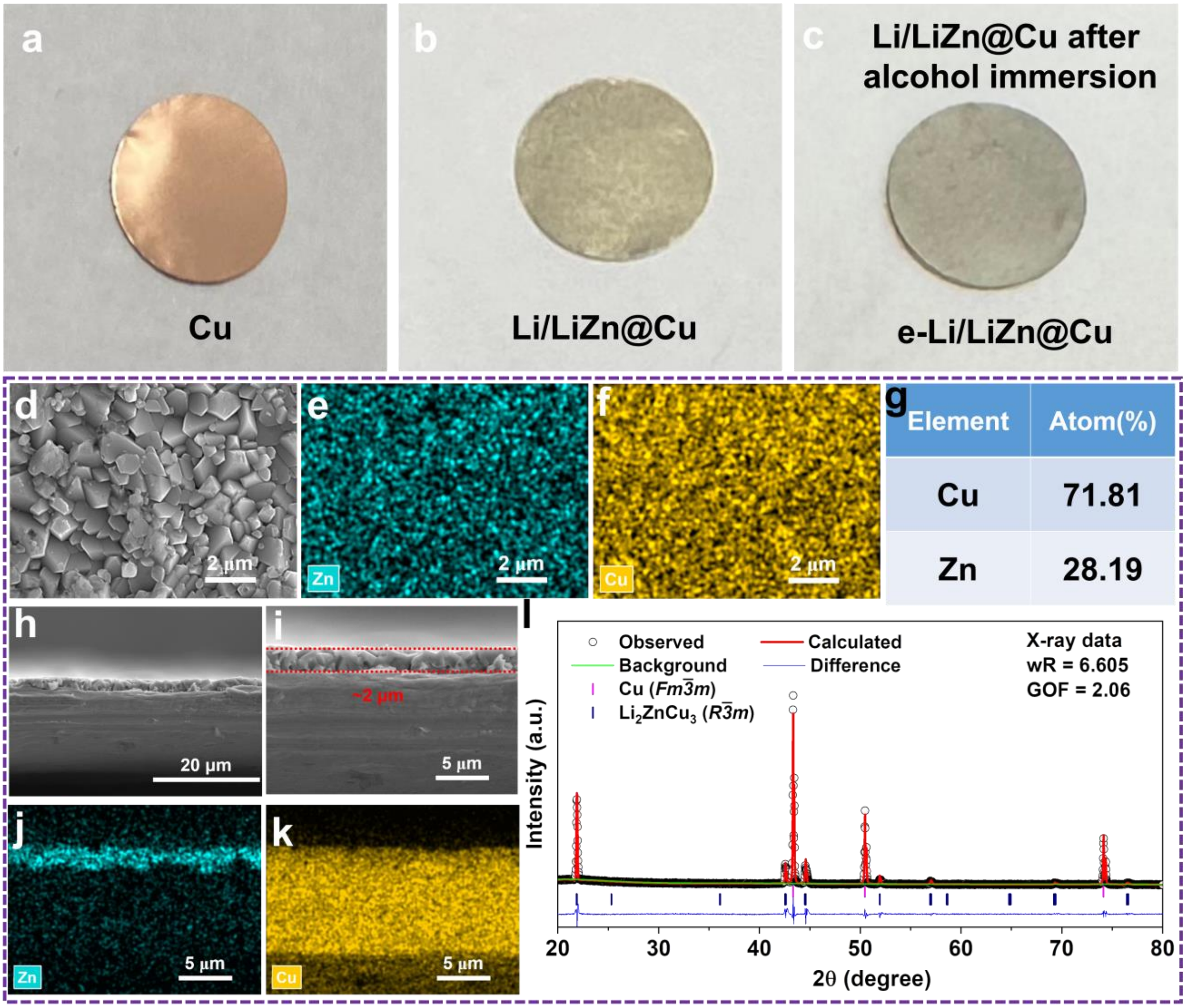

Supplementary Fig. 17. Optic images of the (a) Cu foil, (b) Li/LiZn@Cu, and (c) Li/LiZn@Cu after alcohol immersion (e-Li/LiZn@Cu). (d) Top-view SEM images, (e-f) the corresponding EDS mapping, and (g) results of e-Li/LiZn@Cu. (h-i) Cross-section SEM images and (j-k) the corresponding EDS mapping of e-Li/LiZn@Cu. (l) Rietveld refinement of XRD pattern of the e-Li/LiZn@Cu sample.

Supplementary Table 3. Rietveld refinement results of XRD data for the e-Li/LiZn@Cu sample.

Overall composition: Li_2ZnCu_3 (conventional cell). Space group: $R\bar{3}m$, No. 166 Lattice constants: a = b = 4.974 Å, c = 12.212 Å, V = 261.634 $Å^3$, α = 90º, β = 90º, γ = 120º					
atom	site	x	y	z	Frac.
Li	6c	0	0	-0.32994	1
Zn	3b	0	0	-0.5	1
Cu	9e	-0.16667	0.16667	-0.33333	1
Overall composition: Cu. Space group: $Fm\bar{3}m$, No. 225 Lattice constants: a = b = c = 3.615 Å, V = 47.259 $Å^3$					
atom	site	x	y	z	Frac.
Cu	4a	0	0	0	0.912

The e-Li/LiZn@Cu electrode is obtained by immersing and rinsing with the alcohol solution. After the drying process, as shown in Supplementary Fig. 17a-c, differing from the red-orange color of the pristine Cu or silver color of the Li/LiZn@Cu, the sample after the detachment of Li/LiZn from Cu foil shows a different surface color. Due to the direct contact between the Cu surface and molten Li-Zn mixture, the re-alloying reaction and Li_2ZnCu_3 alloy mainly exist on the Cu surface. Moreover, the top-view SEM image of the e-Li/LiZn@Cu also displays a pyramid-shaped morphology, which is completely different from Cu foil (Supplementary Fig. 17d), indicating the reconstruction of the smooth Cu surface by re-alloying reaction. The uniform distribution of Zn and Cu elements with a close atomic ratio of 1:3 between Zn and Cu evidences that the surface composition of e-Li/LiZn@Cu is Li_2ZnCu_3 alloy (Supplementary Fig. 17e-g). The cross-section SEM and corresponding EDS mapping images indicate that the thickness of Li_2ZnCu_3 is ~ 2 μm (Supplementary Fig. 17h-k). The pronounced Li_2ZnCu_3 peaks of the Rietveld refinement of the XRD pattern of the e-Li/LiZn@Cu sample further verifies the preservation of the Li_2ZnCu_3 layer on the Cu surface (Supplementary Fig. 17l and Table 3). Based on the above characterizations and analysis, the Li_2ZnCu_3 alloy mainly presents at the interface between the Li/LiZn layer and Cu substrate, not inside the Li/LiZn layer.

Supplementary Table 4. Detailed parameters of the pouch cell (stacked number of layers: 10) coupled with commercial NCM811 cathode and different thicknesses of Li metal anodes. Except for the parameters of Li metal anodes with different thicknesses, other parameters refer to Small 2023, 19, 2205653[1].

Composition	Parameter	Value
Cathode (double side)	Areal mass loading of each-side NCM811	18 mg cm^{-2}
	Areal mass loading of Al	3.2 mg cm^{-2}
	Total areal weight of cathode	392 mg cm^{-2}
	Areal capacity of each-side NCM811	3.11 mAh cm^{-2}
	Total areal capacity	62.2 mAh cm^{-2}
	Average voltage	3.8 V
Separator	Total areal weight of separator	16 mg cm^{-2}
Electrolyte (3g Ah^{-1})	Total areal weight of electrolyte	186.6 mg cm^{-2}
Package foil and tabs	Total areal weight of package foil and tabs	24 mg cm^{-2}
Pouch cell	Total areal weight (anode not included, $W_{T\text{-}A}$)	618.6 mg cm^{-2}
	Total areal energy (E_T)	236.36 Wh cm^{-2}

Detailed calculation process

The volumetric energy density of Li:

$$3860 \text{ mAh g}^{-1} \times 0.534 \text{ g cm}^{-3} = 2061.24 \text{ mAh cm}^{-3}$$

The thickness of 1 mAh cm^{-2} Li:

$$\frac{1 \text{ mAh cm}^{-2}}{2061.24 \text{ mAh cm}^{-3}} = 4.85\ \mu\text{m}$$

Take Li metal anode with a maximum thickness of 400 μm as an example. The areal capacity is

$$\frac{400}{4.85} \text{mAh cm}^{-2} = 82.47 \text{ mAh cm}^{-2}$$

For the waste rate of Li resource (R_w, when N/P=0, the R_w is 0), the R_w is defined as follows

$$R_w = \frac{n \times 3.11}{82.47}$$

where the n is the value of the applied N/P ratio.

Thus, the R_w of 400 μm-thick Li metal anode is 100%, while the value is 11.3% when the N/P ratio is 3 (corresponding to 45.3 μm-thick Li metal anode).

The areal mass loading of Li metal anode with 400 μm:

$$\frac{82.47\ \text{mAh cm}^{-2}}{3860\ \text{mAh g}^{-1}} = 21.37\ \text{mg cm}^{-2}$$

To assemble the pouch cell with 10 stacked layers, the number of needed double-side Li metal anodes is nine, while the number of single-side is two, in which the areal mass loading of Cu current collector is 7.2 mg cm^{-2} (8 μm).[1] Thus, the total areal weight of the anode (W_A) is

$$W_A = (21.37\ \text{mg cm}^{-2} \times 2 + 7.2\ \text{mg cm}^{-2}) \times 9 + (21.37\ \text{mg cm}^{-2} + 7.2\text{mg cm}^{-2}) \times 2 = 506.6\ \text{mg cm}^{-2}$$

The total areal weight (W_T) of the pouch cell:

$$W_T = W_A + W_{T-A} = 1125.2\ \text{mg cm}^{-2}$$

For gravimetric energy density, the value is

$$\frac{E_T}{W_T} = 210\ \text{Wh Kg}^{-1}$$

If the thickness of the Li metal anode can be further reduced to less than 50 μm, the corresponding N/P ratio is ~3.3, close to the ideal value of less than 3. At that time, the energy density of the commercial pouch cell can increase to more than 315 Wh kg^{-1}. The energy density can be further enhanced after increasing the stacked layers and optimizing the weight of the electrolyte, cathode, current collector, etc.

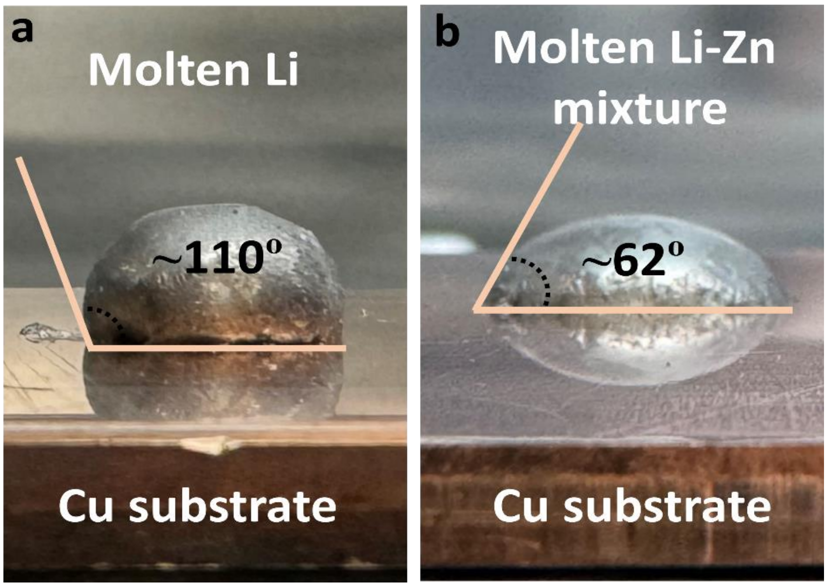

Supplementary Fig. 18. Optic photos of the contact angles of the (a) molten Li and (b) Li/LiZn on Cu substrate.

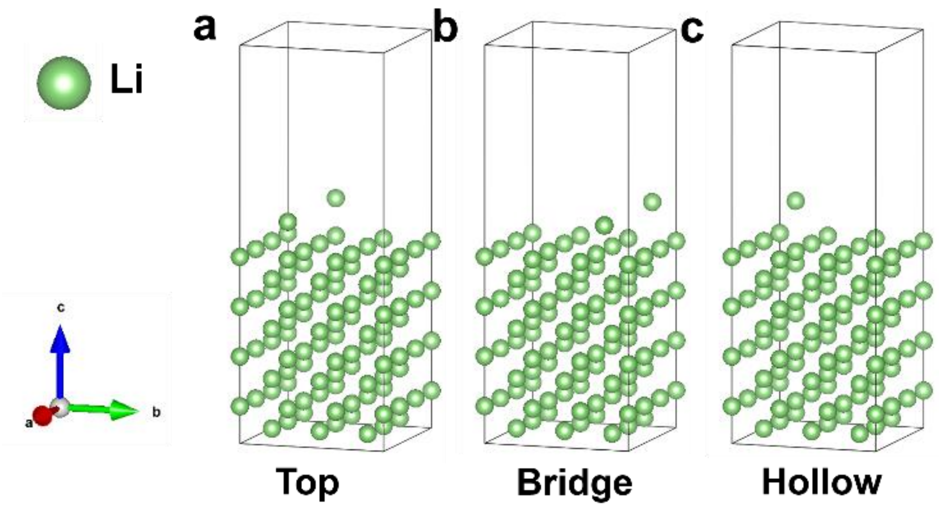

Supplementary Fig. 19. Different possible adsorption sites on the Li (100): (a) Top; (b) Bridge; (c) Hollow.

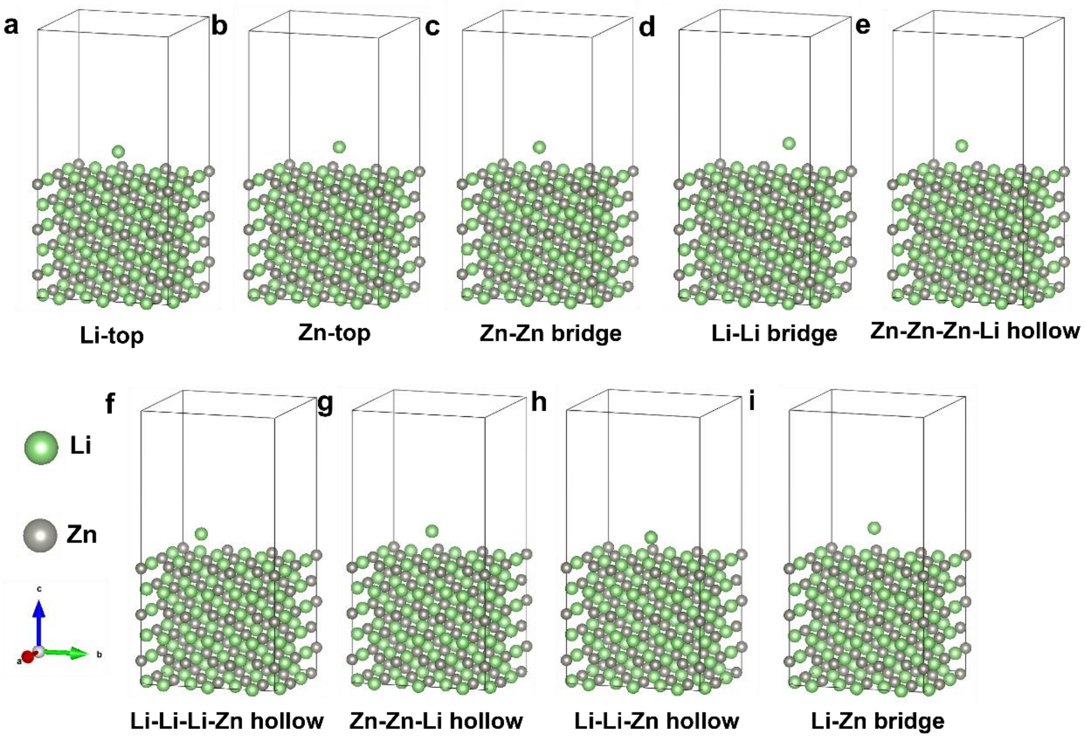

Supplementary Fig. 20. Different possible adsorption sites on the LiZn (110): (a) Li-top; (b) Zn-top; (c) Zn-Zn bridge; (d) Li-Li bridge; (e) Zn-Zn-Zn-Li hollow; (f) Li-Li-Li-Zn hollow; (g) Zn-Zn-Li hollow; (h) Li-Li-Zn hollow; (i) Li-Zn bridge.

Supplementary Table 5. Calculated surface energies of Li_2ZnCu_3.

Composition	Surface miller indices	Surface energy (J m^{-2})
Li_2ZnCu_3	(001)	0.694
	(100)	0.852
	(110)	0.924
	(111)	0.952

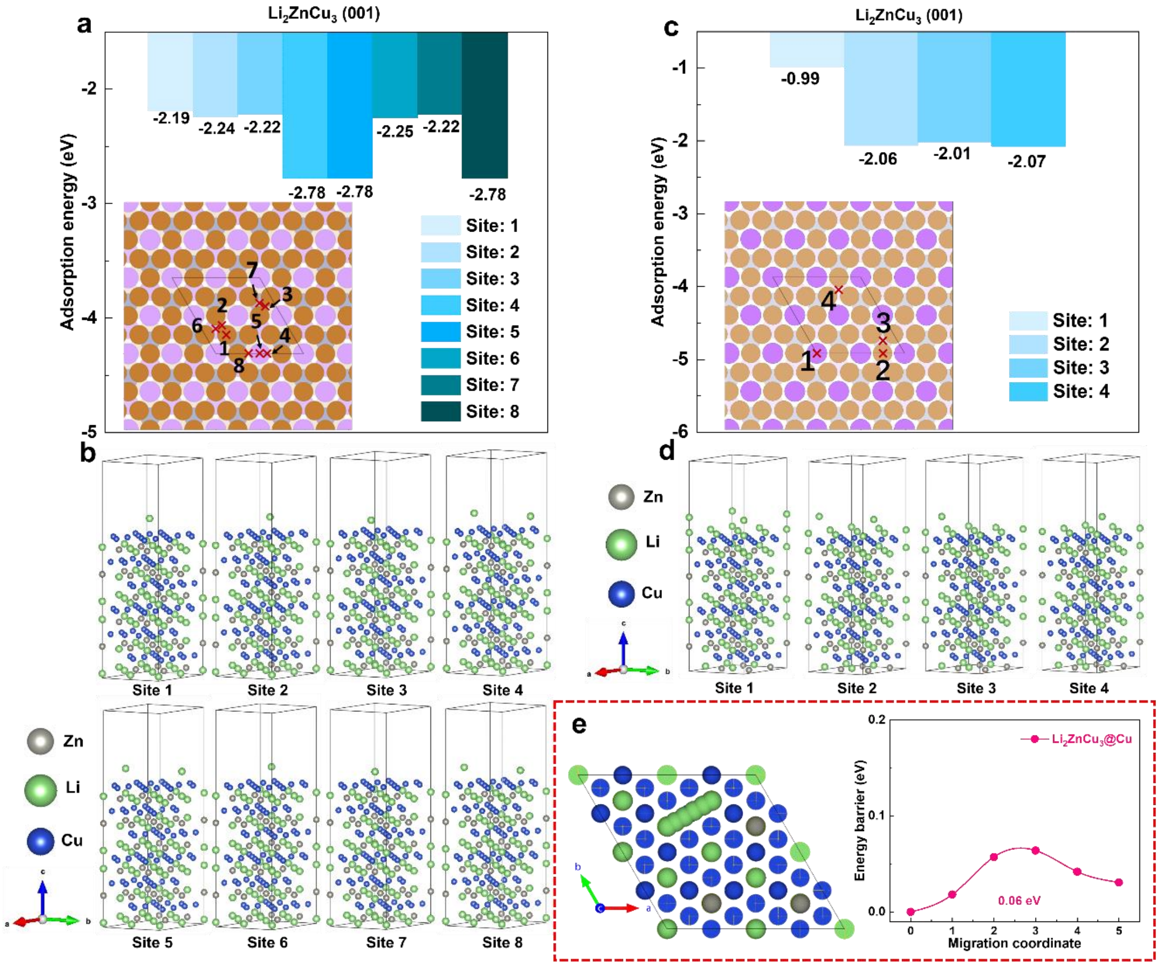

Supplementary Fig. 21. (a) Li absorption energies on the Li_2ZnCu_3 (001) surface and (b) corresponding adsorption sites. (c) Another configuration of Li absorption energies on the Li_2ZnCu_3 (001) surface and (d) corresponding adsorption sites. (e) The diffusion barriers of Li along the Li_2ZnCu_3 (001) surface.

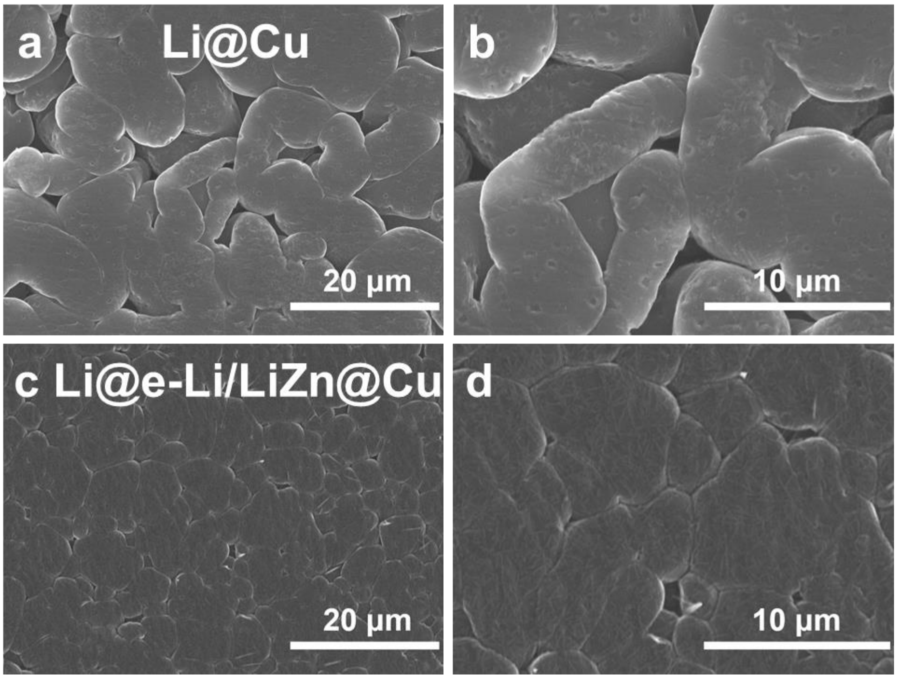

Supplementary Fig. 22. Top-view SEM images of (a-b) Li@Cu and (c-d) Li@e-Li/LiZn@Cu electrodes after Li deposition at 3 mAh cm^{-2}.

Compared with Li and LiZn, few literature has touched upon Li_2ZnCu_3. The Li_2ZnCu_3 (001) is selected as the stable surface for model establishment due to its lowest surface energy (Supplementary Table 5). As shown in Supplementary Fig. 21a-d, the Li absorption energy of Li_2ZnCu_3 (001) surface is -2.78 eV (Li_2ZnCu_3 (001) surface possesses two possible adsorption structures towards Li), which is even more than LiZn (110) (-2.07 eV), let alone compared to Li (100). It suggests that Li_2ZnCu_3 alloy possesses a stronger Li^+ ion capture ability, which can effectively promote Li nucleation and subsequently suppress the uneven Li deposition. However, as shown in Supplementary Fig. 21e, Li_2ZnCu_3 alloy does not have significant advantages in promoting Li surface diffusion compared to LiZn alloy (0.03 eV) and bare Li (0.06 eV), due to the same value (0.06 eV) of surface diffusion barrier along the Li_2ZnCu_3 (001) surface with Li (100). Consequently, compared with bare Li, Li_2ZnCu_3 alloy can improve the uniform Li deposition to some extent due to its superior lithiophilicity, which can be further proved by the deposition morphology comparisons (plating 3 mAh cm^{-2} Li) of Li@Cu (prepared by electrochemical plating, Supplementary Fig. 22a-b) and Li@e-Li/LiZn@Cu (Li deposited e-Li/LiZn@Cu electrode, Supplementary Fig. 22c-d).

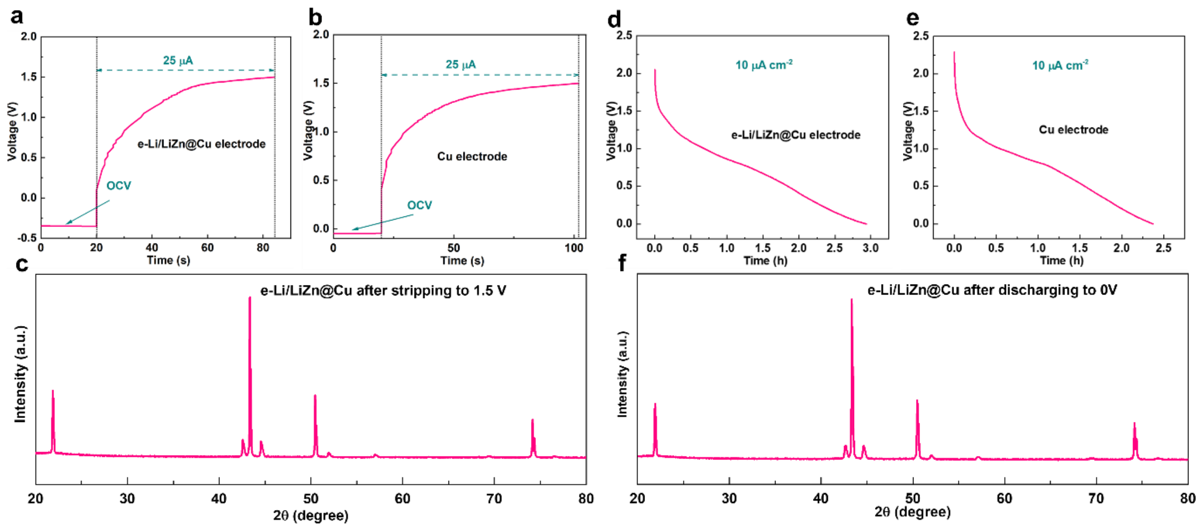

Supplementary Fig. 23. Voltage profiles of (a) e-Li/LiZn@Cu||Cu and (b) Cu||Cu cells after charging to 1.5 V at 25 μA. (c) XRD patterns of the dissembled e-Li/LiZn@Cu electrodes after stripping to 1.5 V. Voltage profiles of (d) e-Li/LiZn@Cu||Cu and (e) Cu||Cu cells after discharging to 0 V at 10 $\mu A\ cm^{-2}$. (f) XRD patterns of the dissembled e-Li/LiZn@Cu electrodes after discharging to 0 V.

The phase stability of Li_2ZnCu_3 alloy during the electrochemical process is explored based on the e-Li/LiZn@Cu electrode. Firstly, the e-Li/LiZn@Cu||Cu (i.e. working electrode: e-Li/LiZn@Cu; counter electrode: Cu) and Cu||Cu cells were assembled to verify the stability during the Li charging process. A low constant charging current of 25 μA is applied for Li^+ ion extraction from the Li_2ZnCu_3 alloy as much as possible. As shown in Supplementary Fig. 23a-b, the voltage profiles of e-Li/LiZn@Cu||Cu and Cu||Cu cells are similar, both rapidly rising to 1.5 V in a short time, which indicates that Li^+ ions can not be dealloyed from the Li_2ZnCu_3 alloy. Interestingly, such voltage profiles demonstrate the capacitive behavior. This capacitive ion storage behavior is a non-Faradic process, and its current response is basically independent of the battery.[2] Moreover, the half cells with Li||e-Li/LiZn@Cu and Li||Cu configurations were further assembled to verify the stability of Li_2ZnCu_3 alloy during discharging process. The Li||e-Li/LiZn@Cu cell also shows a similar voltage profile to the Li||Cu cell, in which the platform of voltage drop can be ascribed to the decomposition of electrolyte and formation of SEI (Supplementary Fig. 23d-e). Furthermore, as shown in Supplementary Fig. 23c,f, all disassembled electrodes do not significantly change in XRD patterns compared to the pristine e-Li/LiZn@Cu (Supplementary Fig. 17l).

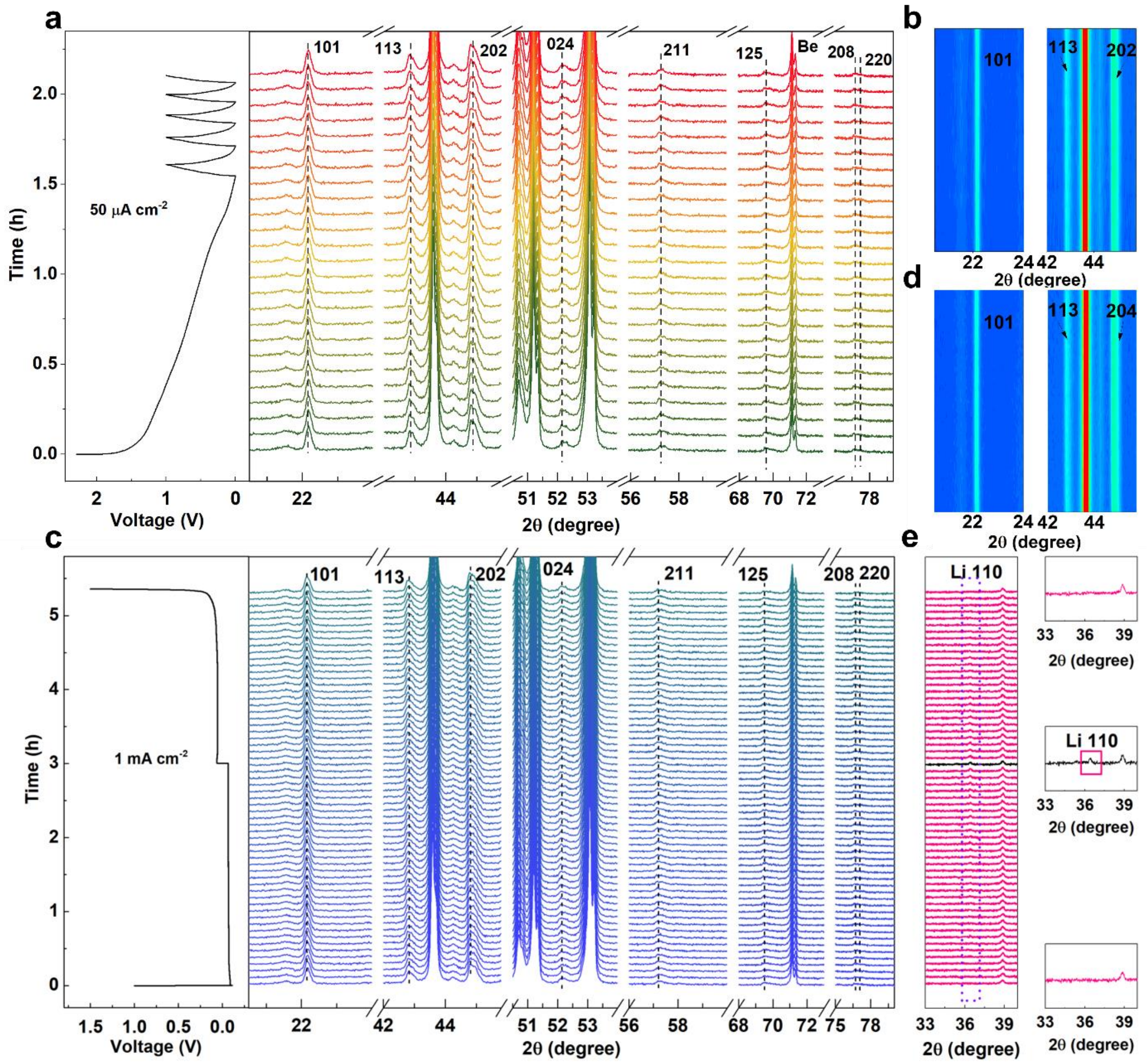

Supplementary Fig. 24. (a) *In-situ* XRD analysis of e-Li/LiZn@Cu electrode and (b) the corresponding contour maps during Li discharging and charging with a voltage range from 0 to 1 V at 50 μA cm^{-2}. (c) *In-situ* XRD analysis of e-Li/LiZn@Cu electrode, (d) the corresponding contour maps, and (e) selected region of Li peaks during Li plating with a fixed Li amount of 3 mA cm^{-2} and stripping to 1.5 V at 1mA cm^{-2}.

The *in-situ* Li||e-Li/LiZn@Cu cell is further assembled to study the electrochemical stability of Li_2ZnCu_3 alloy in depth. A thinner Cu foil (6 μm) was used as the substrate for better X-ray diffraction observation. The whole testing process can be divided into two parts, one of which is the Li discharging and charging with a voltage range from 0 to 1 V at 50 μA cm^{-2} and the other of which involves the Li plating with a fixed Li amount of 3 mA cm^{-2} and stripping to 1.5 V at 1mA cm^{-2}. As shown in

Supplementary Fig. 24a-b, the peak intensities and positions belonging to Li_2ZnCu_3 alloy are highly consistent without peak weakening or shifting during the Li discharging and charging process, indicating that Li_2ZnCu_3 is electrochemical inactive within the above-operated voltage. Moreover, the Li_2ZnCu_3 also maintains a stable phase throughout the Li plating and stripping process, even when the cut-off voltage is up to 1.5 V (Supplementary Fig. 24c-d). According to the above comprehensive XRD and electrochemical analysis, it can be concluded that the Li_2ZnCu_3 alloy is an inert alloy during the electrochemical tests, just like the Cu substrate, so that will not participate in electrochemical cycling. In addition, a shallow peak assigned to the Li (110) plane gradually emerges during Li plating and fades after Li stripping (Supplementary Fig. 24e). Such a low signal of Li can be attributed to the low content on the Cu substrate. Due to the excellent electrochemical stability of the Li_2ZnCu_3 alloy and to reduce the interference of the Cu substrate on LiZn and Li characteristic peaks to better clarify the phase changes of the Li/LiZn layer during the Li stripping/plating process, the working electrode of in-situ XRD characterization in Fig. 3j is set using the Li/LiZn anode for simplification.

Supplementary Table 6. Comparison of the basic features and cycling performance with various reported Li metal batteries

Anode			Symmetric cell			Full cell							Ref.
						Cathode		N/P ratio	Cycling performance				
**Recipe**	**Total capacity**	**Thickness**	**Cycled capacity/Applied current density**	**Depth of discharge (the ratio between the cycled capacity and total capacity)**	**Lifespan**	**Composition**	**Capacity**		**Rate**	**Lifespan**	**Cycling voltage range**	**Capacity retention**	
LV-Li	>50 mAh cm^{-2}	~1000 µm	6 mA cm^{-2}/3 mAh cm^{-2}	<6%	300 h	LFP	~5 mAh cm^{-2}	>10	0.5 C	150 cycles	2.5-4.0 V	~94.5%	**3**
PDDA-TFSI@Li	19.39 mAh cm^{-2}	100 µm	1 mA cm^{-2}/1 mAh cm^{-2}	~5.2%	1000 h	LFP	~2.8 mAh cm^{-2}	~6.9	0.5 C	200 cycles	2.5-4.0 V	97.5%	**4**
SLCN	56.36 mAh cm^{-2} (0.0146 g cm^{-2} Li)	290 µm	1 mA cm^{-2}/1 mAh cm^{-2}	~1.8%	2000 h	S/C composites	~1.27 mAh cm^{-2}	~44.4	0.5 C	200 cycles	1.7-2.8 V	72.4%	**5**
Li–Zn	9.9 mAh cm^{-2}	~50 µm	1 mA cm^{-2}/1 mAh cm^{-2}	10%	700 h	LFP	3 mAh cm^{-2}	3.3	0.5 C	130 cycles	2.8-3.8 V	~87%	**6**
GaLi–Li	>32 mAh cm^{-2}	>160 µm	2 mA cm^{-2}/2 mAh cm^{-2}	<6.25%	300 h	LFP	1.9 mAh cm^{-2}	>16.8	0.5 C	200 cycles	2.4-3.8 V	93.6%	**7**
LaF$_3$-Li	20 mAh cm^{-2}	/	/	/	/	NCM811	~4 mAh cm^{-2}	5	1 C	200 cycles	3.0-4.3 V	87.66%	**8**
SbF$_3$@Li	~20 mAh cm^{-2}	~100 µm	0.2 mA cm^{-2}/0.2 mAh cm^{-2}	~1%	310 h	LFP	~0.34 mAh cm^{-2}	~58.8	0.2 C	200 cycles	2.5-4.0 V	94.5%	**9**
AFH-25	~51.5 mAh cm^{-2}	250 µm	1 mA cm^{-2}/1 mAh cm^{-2}	~2%	850 h	NCM111	~1.3 mAh cm^{-2}	~39.6	1 C	150 cycles	2.7-4.2 V	80.01%	**10**
xPCMS-g-PEGMA/LN@Li	~93 mAh cm^{-2}	450 µm	5 mA cm^{-2}/1 mAh cm^{-2}	~1.1%	9400 h	LFP	~2 mAh cm^{-2}	~46.5	0.2 C	100 cycles	2.0-4.0 V	94%	**11**
ZeroVE-Li	13.2 mAh cm^{-2}	104 µm	1 mA cm^{-2}/1 mAh cm^{-2}	~7.6%	1500 h	NMC811	3.7 mAh cm^{-2}	3.6	0.54 C	200 cycles	/	63%	**12**
Li-MnO$_x$/CNT	1.67 mAh cm^{-2}	10 µm	0.5 mA cm^{-2}/0.3 mAh cm^{-2}	~18.0%	475 h	LCO	~0.4 mAh cm^{-2}	4	0.5 C	150 cycles	3.0-4.35 V	80.3%	**13**
Li@eGF	3.68 mAh cm^{-2}	20 µm	/	/	/	LFP	3.24 mAh cm^{-2}	~1.14	0.5 C	200 cycles	3.0-4.0 V	81%	**14**
Li/LiZn@Cu	4.4 mAh cm^{-2}	35 µm				LFP	1.3 mAh cm^{-2}	~3.4	0.5 C	230 cycles	2.4-4.2 V	98.0%	**Our work**
Li/LiZn@Cu	4.4 mAh cm^{-2}	35 µm				LFP	2 mAh cm^{-2}	~2.2	0.5 C	130 cycles	2.4-4.2 V	90.1%	**Our work**
Li/LiZn@Cu	4.4 mAh cm^{-2}	31 µm	0.5 mA cm^{-2}/1 mAh cm^{-2}	~22.7%	690 h	LCO	1.8 mAh cm^{-2}	~2.5	0.5 C	125 cycles	2.8-4.5 V	74%	**Our work**
Li/LiZn@Cu	4.4 mAh cm^{-2}	31 µm	1 mA cm^{-2}/1 mAh cm^{-2}		1200 h	LCO (pouch cell)	3.27 mAh cm^{-2}	~1.35	0.1 C	40 cycles	2.8-4.5 V	90% (compared with the highest capacity)	**Our work**

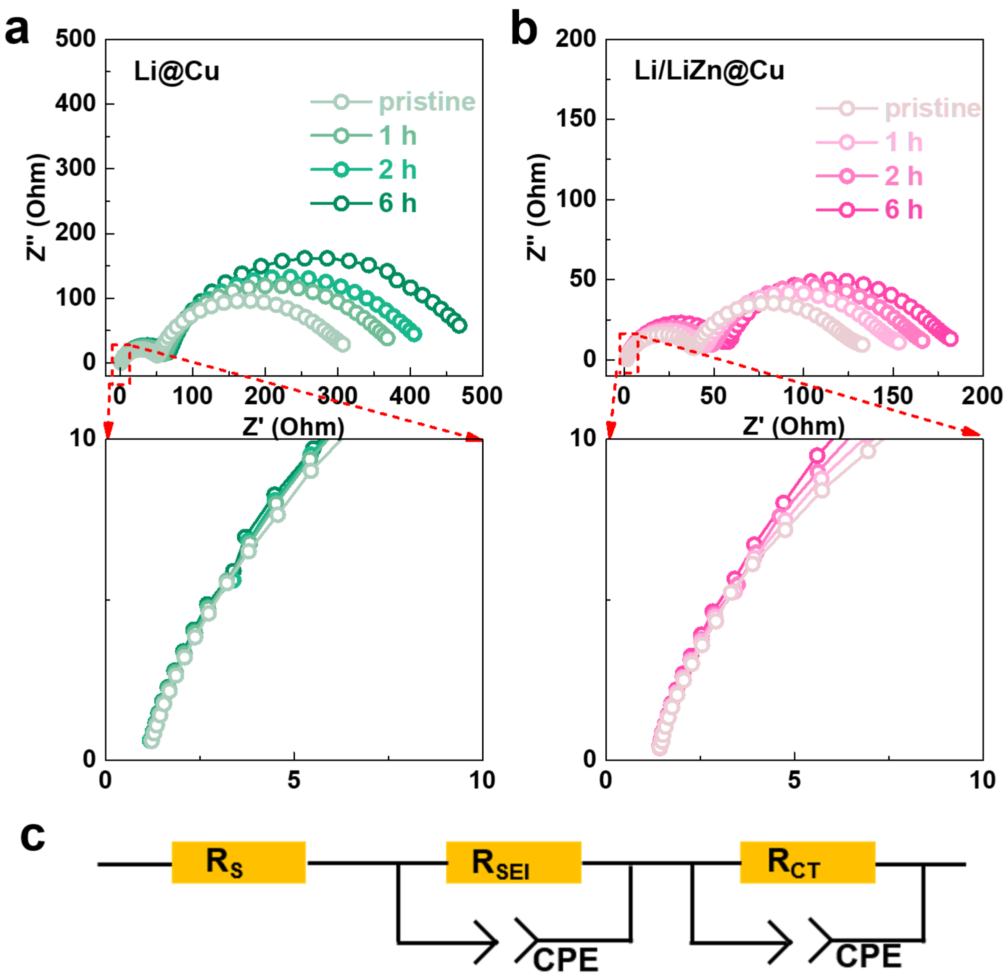

Supplementary Fig. 25. EIS spectra of (a) Li@Cu and (b) Li/LiZn@Cu symmetric cells with the different open-circuit times before cycling and the corresponding (c) equivalent circuit. R_S: series resistance; R_{SEI}: SEI resistance; R_{CT}: charge-transfer resistance; CPE: constant phase element.

Supplementary Table 7. The fitting data of Supplementary Fig. 25.

**Time**	**Li@Cu**			**Li/LiZn@Cu**		
	R_S (Ω)	R_{SEI} (Ω)	R_{CT} (Ω)	R_S (Ω)	R_{SEI} (Ω)	R_{CT} (Ω)
**Pristine**	1.067	50.23	253.9	1.333	37.24	91.57
**1 h**	1.043	58.11	311.0	1.314	43.03	106.5
**2 h**	1.026	60.84	346.2	1.296	47.58	116.2
**6 h**	1.012	64.66	410.7	1.294	55.13	124.3

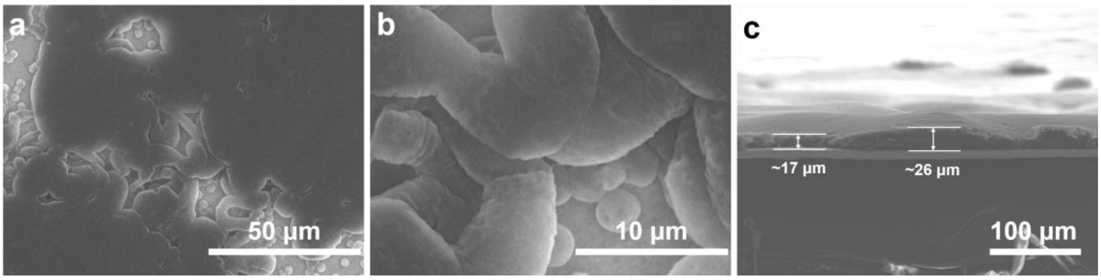

Supplementary Fig. 26. (a-b) Top-view and (c) cross-section SEM images of Li@Cu electrode.

As shown in Supplementary Fig. 25, compared with Li/LiZn@Cu, the Li@Cu possesses higher SEI resistance (representing the diameter value of the semicircle in the high-frequency region) and charge-transfer resistance (representing the diameter value of the semicircle in the low-frequency region) and increases greatly with the increase of open-circuit time (Supplementary Table 7). It indicates that the Li/electrolyte interface of Li@Cu is much more unstable than Li/LiZn@Cu. Due to the inferior native SEI of Li@Cu caused by initially uneven morphology and thickness (Supplementary Fig. 26), more side reactions and poor interface are induced, which can result in a higher overpotential for Li nucleation/deposition at initial cycling. Additionally, such a bad Li/electrolyte interface will influence the subsequent cycling behaviors and lifespan of Li@Cu.

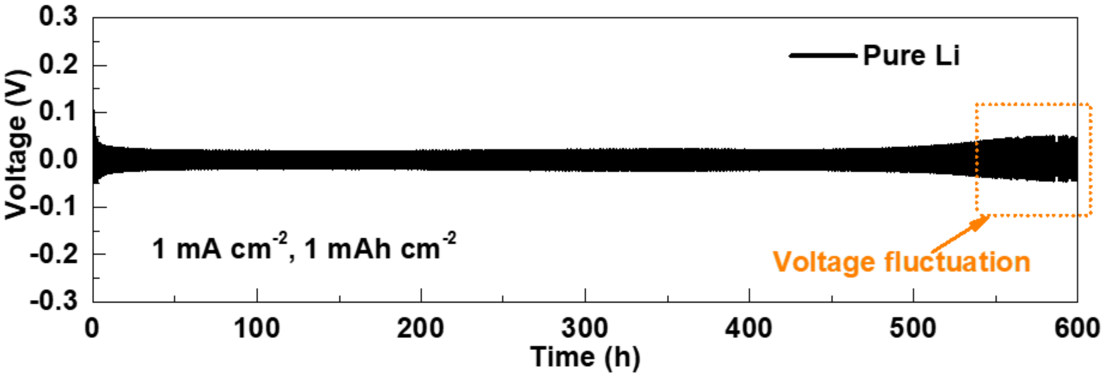

Supplementary Fig. 27. Galvanostatic cycling of the thick Li anode (450 μm) at 1 mA cm^{-2}/1 mAh cm^{-2} in the symmetric cell.

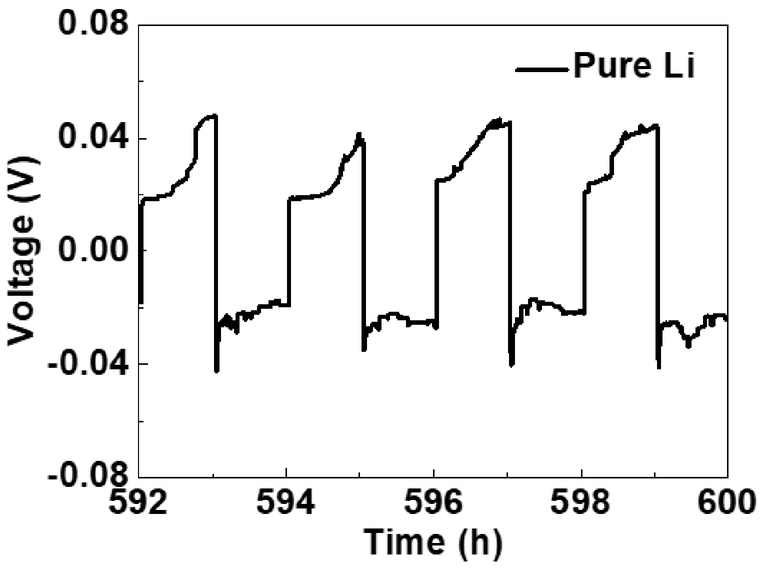

Supplementary Fig. 28. Detailed voltage profile of the thick Li anode (450 μm) at 1 mA cm^{-2}/1 mAh cm^{-2} in the symmetric cell.

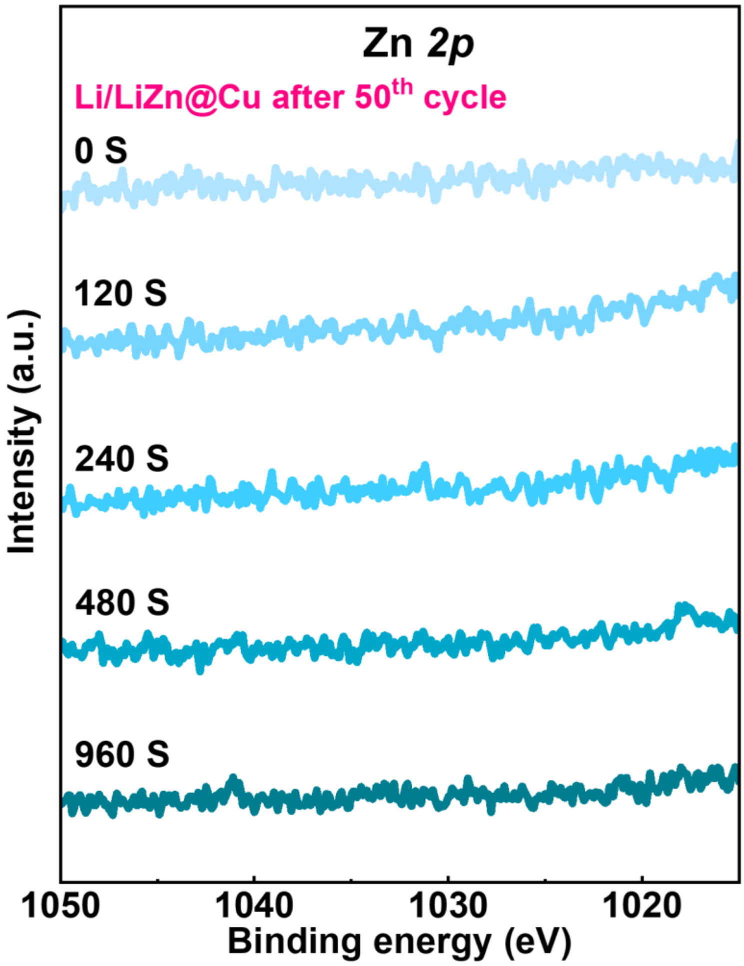

Supplementary Fig. 29. Zn *2p* XPS spectrum of Li/LiZn@Cu anode at different etching times.

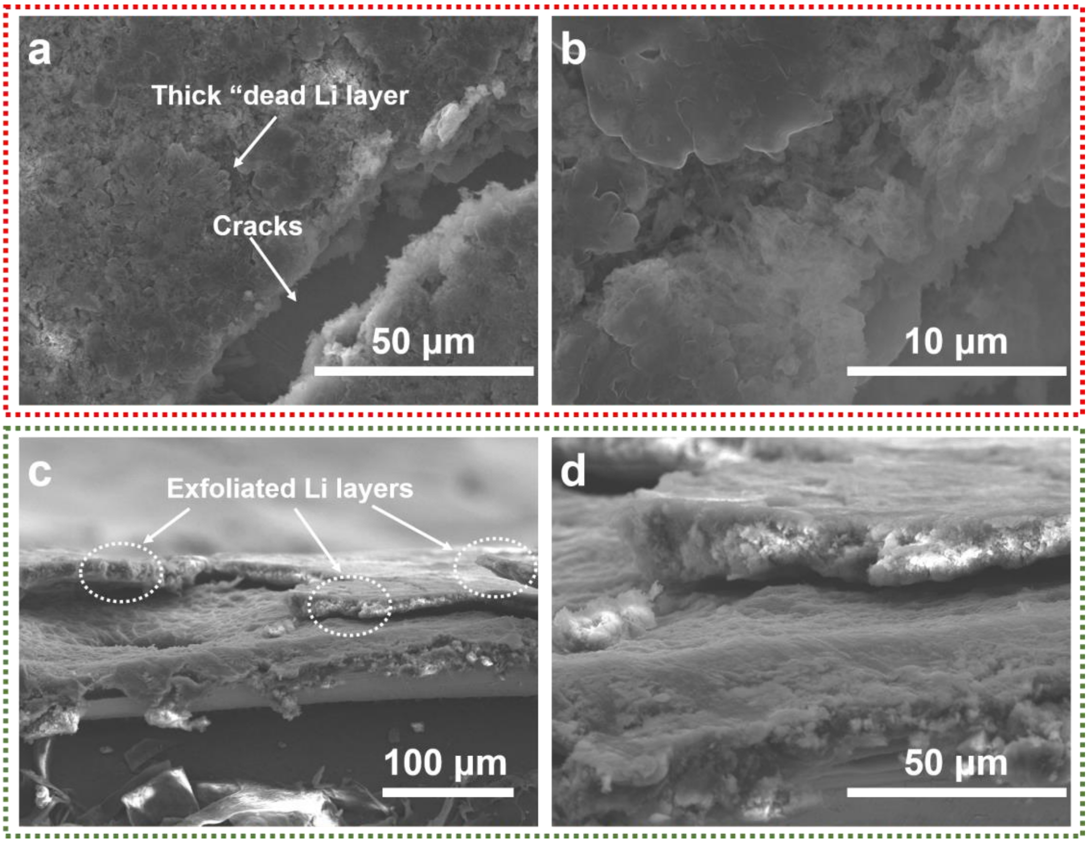

Supplementary Fig. 30. (a-b) Top-view and (c-d) cross-section SEM images of the Li@Cu after the 50th cycle in the symmetric cell.

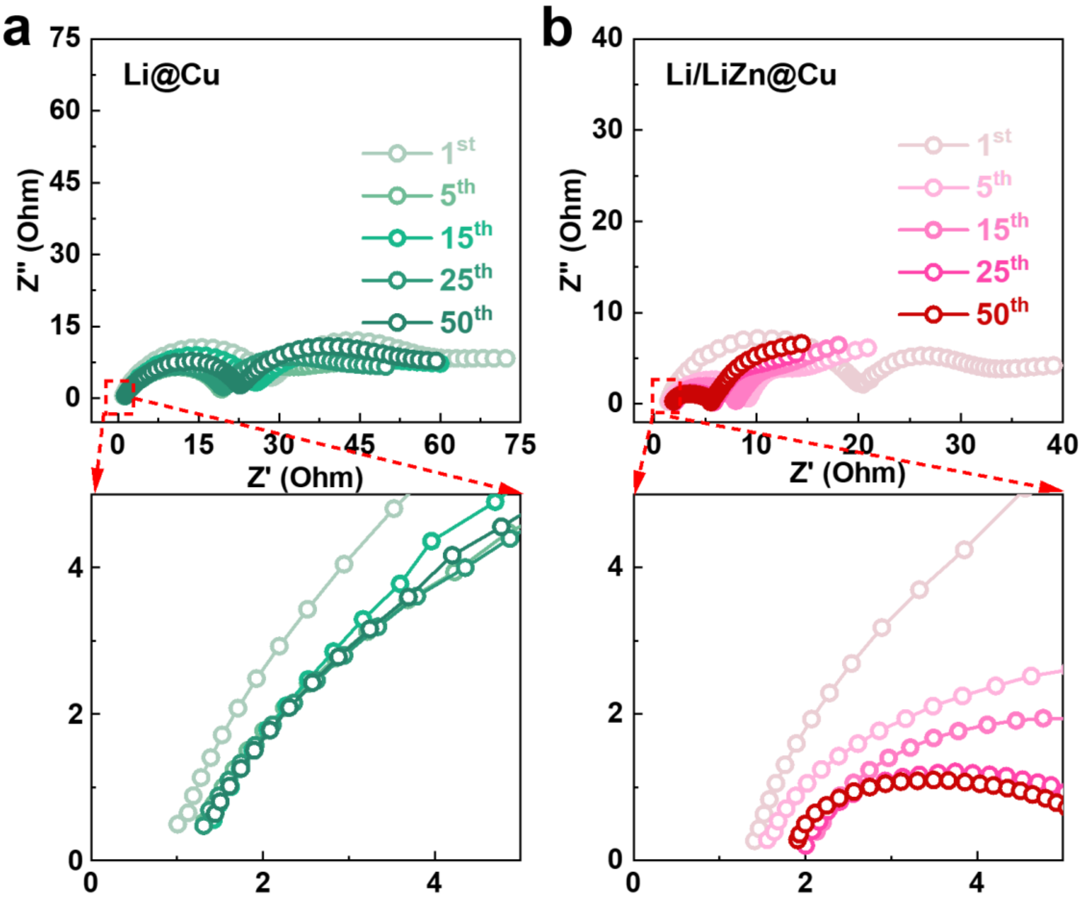

Supplementary Fig. 31. EIS spectra of (a) Li@Cu and (b) Li/LiZn@Cu symmetric cells after different cycle numbers at 1 mA cm^{-2}/1 mAh cm^{-2}.

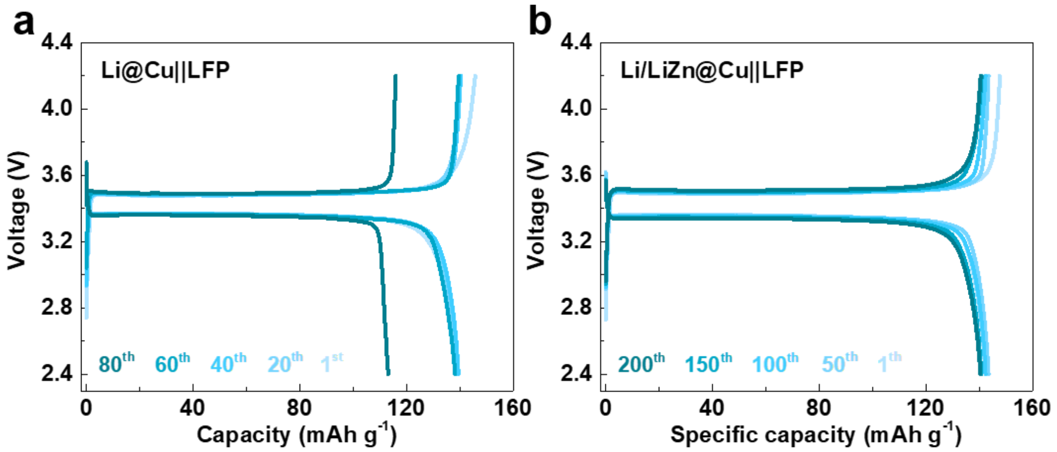

Supplementary Fig. 32. Voltage profiles of the selected cycles for the (a) Li@Cu||LFP and (b) Li/LiZn@Cu||LFP at 0.5 C.

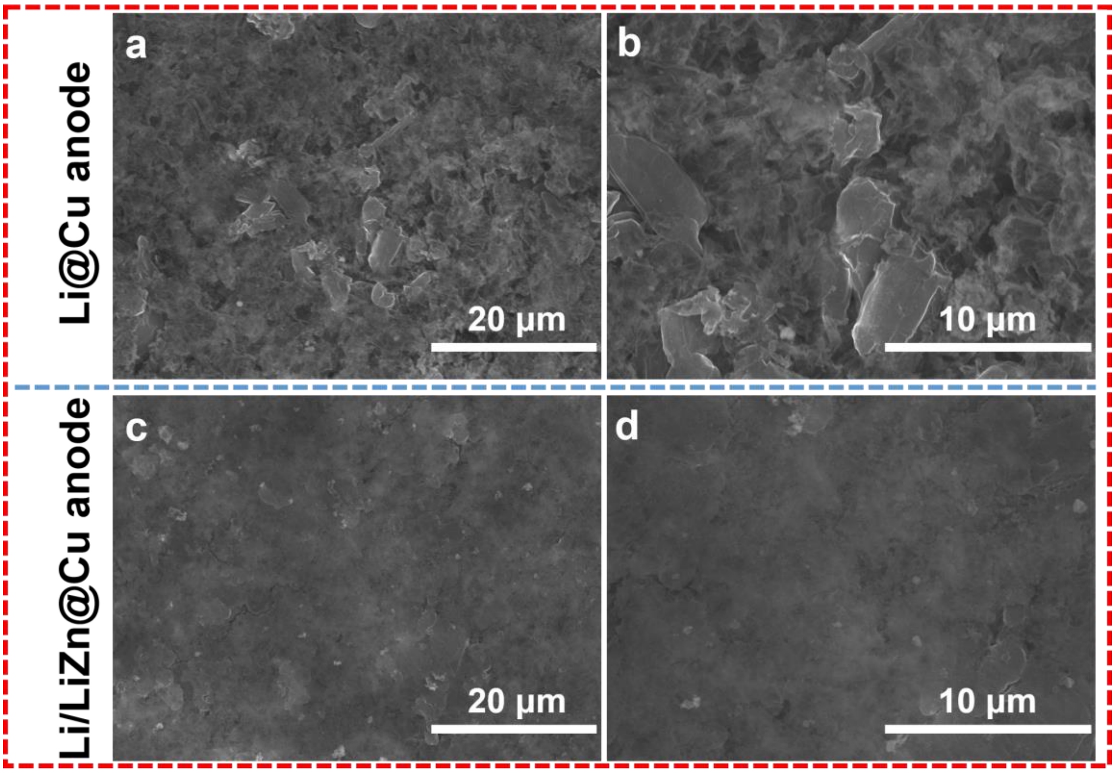

Supplementary Fig. 33. High-magnification SEM images of (a-b) Li@Cu and (c-d) Li/LiZn@Cu anodes after the 30th cycle in the LFP-based full cells.

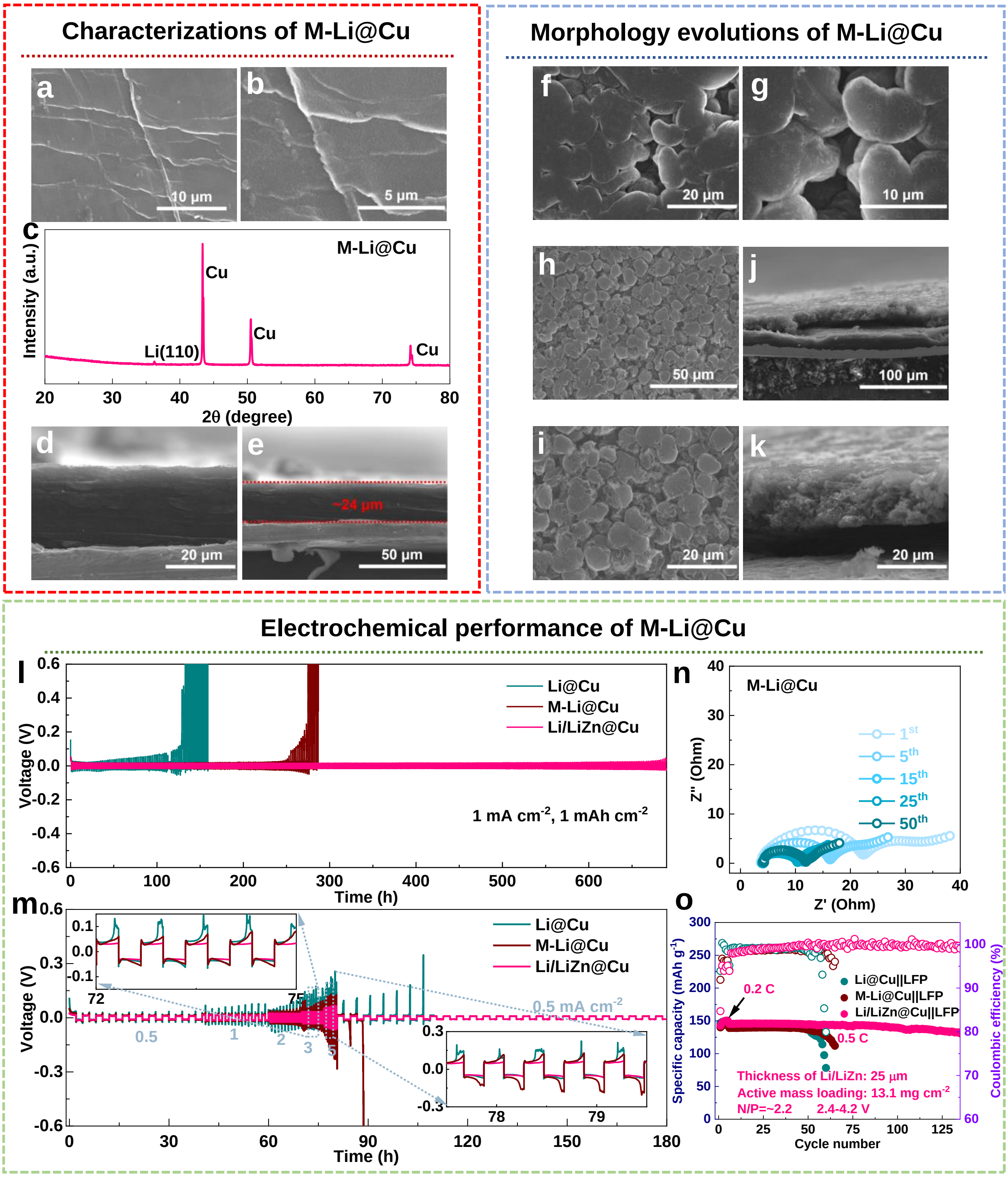

Supplementary Fig. 34. (a-b) Top-view SEM images, (c) XRD pattern, and (d-e) cross-section SEM images of M-Li@Cu. Morphology evolutions of M-Li@Cu after (f-g) plating 3 mAh cm^{-2} Li and (h-i) 50 cycles in symmetric cell at 1 mA cm^{-2}/1 mAh cm^{-2} and (j-k) corresponding cross-section SEM images. Comparisons of the M-Li@Cu-, Li@Cu-, and Li/LiZn@Cu-based symmetric cells in (l) long-term cycling, (m) rate, and (n) EIS tests. (o) Comparisons of the cycling performance of M-Li@Cu-,

Li@Cu-, and Li/LiZn@Cu-based full cells with a high-loading LFP cathode (~2 mAh cm^{-2}).

The electrodeposition-based Li@Cu will be exposed to the electrolyte during fabrication, inevitably introducing unnecessary by-products into the Li anode and making the pre-loss of active Li. To exclude the introduction of rugged Li and "dead Li" in preparation, the mechanical rolling-based thin Li@Cu anode (named the M-Li@Cu and purchased from China Energy Lithium Co., Ltd) is also considered as the control group. As shown in Supplementary Fig. 34a-b, the M-Li@Cu possesses visible mechanical indentations on the surface. The XRD pattern exhibits that the M-Li@Cu only contains the metallic Cu and Li (Supplementary Fig. 34c). The thickness of M-Li@Cu is ~ 34 μm which is similar to the Li/LiZn@Cu electrode (35 μm), in which the bottom region is 10 μm-thick Cu substrate and the top region is 24 μm-thick metallic Li layer (Supplementary Fig. 34d-e). Compared with the Li/LiZn layer, the bare Li layer can not realize the ordered Li growth regulation due to its poor Li adsorption ability and large diffusion barrier for the Li lateral deposition, as verified in Fig. 3a-c. As shown in Supplementary Fig. 34f-g, the surface of the M-Li@Cu electrode presents a Li intertwined morphology with many pores after plating 3 mAh cm^{-2} Li. Such an uneven Li morphology with a high exposure area to electrolyte will bring about excessive SEI formation and active material consumption, accompanied by the aggravated generation of the "dead Li" in subsequent cycles. Consequently, after 50 cycles at 1 mA cm^{-2}/1 mAh cm^{-2} in the symmetric cell with M-Li@Cu||M-Li@Cu configuration, the worse Li morphology can be observed with the partial Li layer detaches from the substrate (Supplementary Fig. 34h-k). This explains the poor long-term cycling performance of the M-Li@Cu||M-Li@Cu cells in Supplementary Fig. 34l, in which the voltage violently fluctuates after only 250 h, whereas the symmetric Li/LiZn@Cu cell delivers a stable voltage platform for 690 h. It is worth mentioning that the cycling performance of M-Li@Cu anode is much better than that of Li@Cu one which is prepared by electrochemical plating. It can be ascribed to the more dense and uniform Li layer on the pristine M-Li@Cu than that of Li@Cu, thereby resulting in a superior Li/electrolyte interface and electrochemical performance than that of the Li@Cu anode in long-term cycles. The EIS analysis also demonstrates this viewpoint. As shown in Supplementary Fig. 34n, although both M-Li@Cu and Li@Cu exhibit an increase in R_{SEI} (the semicircle at high frequency) after 50 cycles, the M-Li@Cu still has a smaller value than that of the Li@Cu (Supplementary Fig. 31a), as well as its better rate

performance in Supplementary Fig. 34m. Furthermore, the M-Li@Cu-based full cell with a high-loading LFP cathode (2 mAh cm^{-2}) was assembled to conduct a systematic evaluation of M-Li@Cu for electrochemical stability. As shown in Supplementary Fig. 34o, the M-Li@Cu||LFP cell delivers a higher capacity retention of ~ 80.0% after 65 cycles than the 54.4% after 60 cycles of Li@Cu||LFP cell, but is much inferior to the Li/LiZn@Cu cell.

Benefiting from the uniform morphology of initial Li and no generation of "dead Li" in the preparation process, the M-Li@Cu exhibits a better electrochemical performance than that of the Li@Cu, no matter in symmetric or full cell configuration. However, the poor Li adsorption and surface diffusion ability of M-Li@Cu also restrict its cycling performance, showing a significant gap compared with the Li/LiZn@Cu electrode.

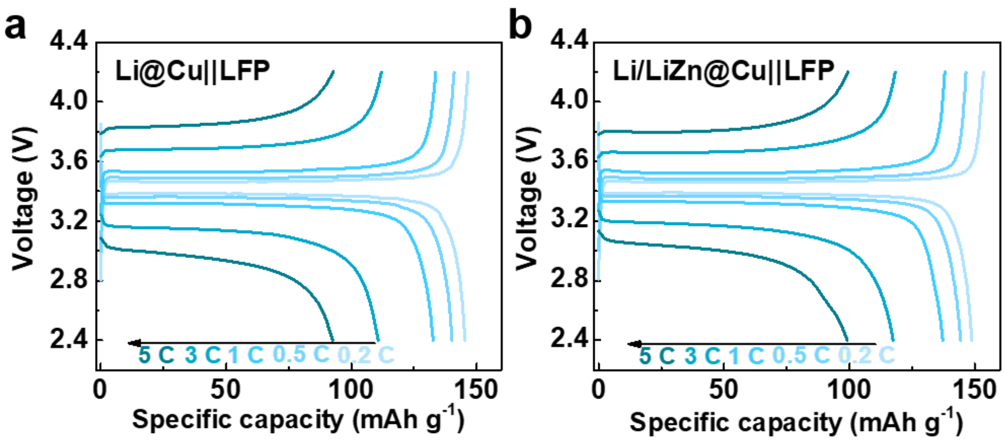

Supplementary Fig. 35. Voltage profiles of the selected cycles for the (a) Li@Cu||LFP and (b) Li/LiZn@Cu||LFP at 0.2, 0.5, 1, 3, and 5 C.

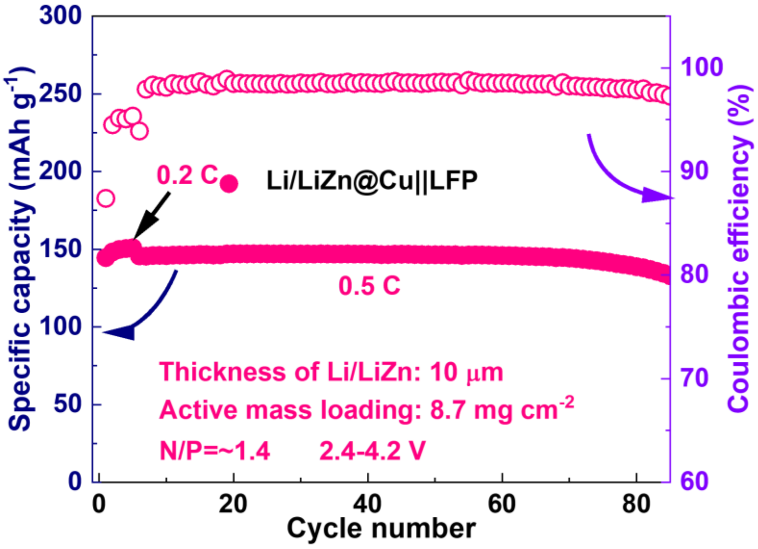

Supplementary Fig. 36. Cycling performance of Li/LiZn@Cu||LFP full cells with an N/P ratio of ~1.4 at 0.5 C.

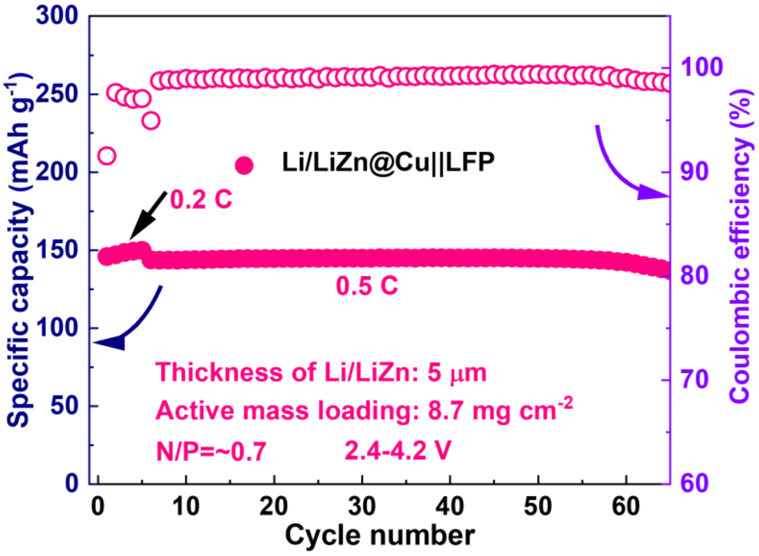

Supplementary Fig. 37. Cycling performance of Li/LiZn@Cu||LFP full cells with an N/P ratio of ~0.7 at 0.5 C.

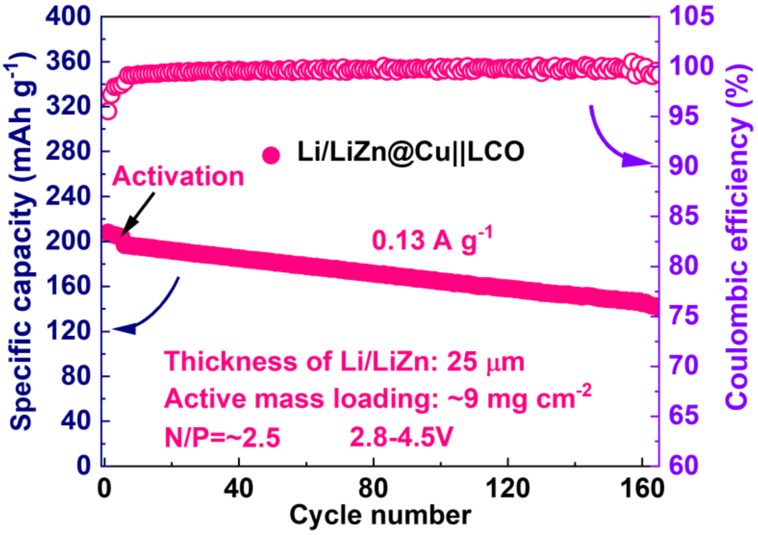

Supplementary Fig. 38. Cycling performance of Li/LiZn@Cu||LCO full cells at 0.13 A g^{-1}.

Supplementary Table 8. Calculated energy density of the Li/LiZn@Cu||LCO coin cell with lean electrolyte.

Component	Value
Li/LiZn@Cu anode	6.9 mg cm^{-2}
LCO cathode (90% active material)	10 mg cm^{-2}
Al foil	3.2 mg cm^{-2}
Electrolyte+Separator	5.6 mg cm^{-2}
Total weight	25.7 mg cm^{-2}
Discharging energy	7.29 mWh cm^{-2}
Gravimetric energy density	283.7 Wh kg^{-1}

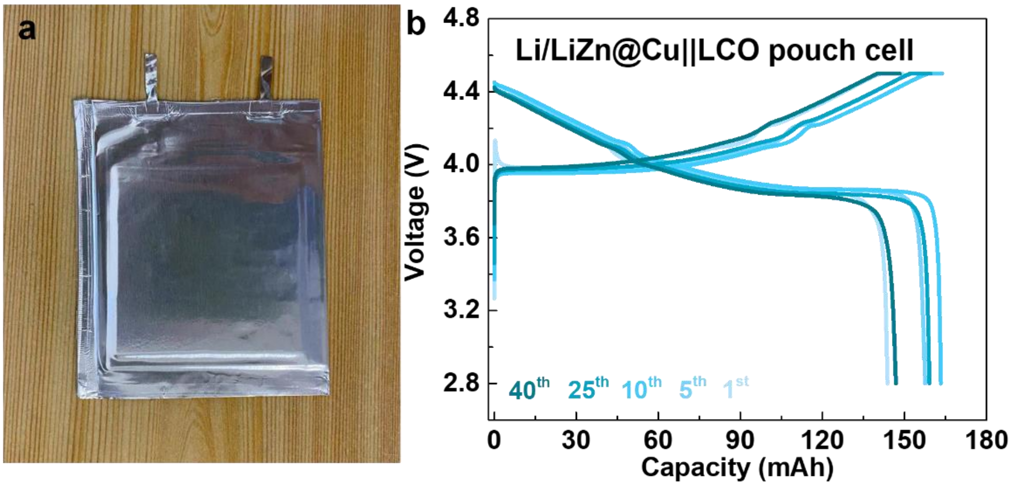

Supplementary Fig. 39. (a) Optic image and (b) corresponding voltage profiles of Li/LiZn@Cu||LCO pouch cell.

Supplementary Table 9. Calculated energy density of the Li/LiZn@Cu||LCO pouch cell with double-side LCO cathode.

Composition	Parameter	Value
LCO cathode (double side)	Active mass loading (91% active material)	35.3 mg cm^{-2} (double side)
	Areal capacity	6.54 mAh cm^{-2}
	Stacked number of layers	1 (double side)
Li/LiZn@Cu	Areal capacity	4.4 mAh cm^{-2} (single layer)
	N/P	1.35
	Thickness	31 μm (single layer, including 6 μm-thick Cu foil)
	Areal weight	6.9 mg cm^{-2} (single layer, including 6 μm-thick Cu foil)
	Stacked number of layers	2 (single layer)
Separator	Areal weight	1 mg cm^{-2}
	Stacked number of layers	2
Al foil	Areal weight	2.7 mg cm^{-2}
Electrolyte	Weight	1.83 g Ah^{-1}
Package foil	Areal weight	23.1 mg cm^{-2} (double layer)
Pouch cell	Discharging energy	26.24 mWh cm^{-2}
	Total weight	92.4 mg cm^{-2}
	Gravimetric energy density	284.0 Wh Kg^{-1}

Supplementary Table 10. Calculated energy density of the proposed Li/LiZn@Cu||LCO pouch cell with 10 stacked layers of double-side LCO cathode.

Composition	Parameter	Value
LCO cathode (double side)	Active mass loading (91% active material)	35.3 mg cm^{-2} (double side)
	Areal capacity	6.54 mAh cm^{-2}
	Stacked number of layers	10 (double side)
Li/LiZn@Cu	N/P	1.35
	Areal capacity	4.4 mAh cm^{-2} (single layer)
	Thickness	31 μm (single layer, including 6 μm-thick Cu foil)
	Areal weight	6.9 mg cm^{-2} (single layer, including 6 μm-thick Cu foil)
	Stacked number of layers	20 (single layer)
Separator	Areal weight	1 mg cm^{-2}
	Stacked number of layers	20
Al foil	Areal weight	2.7 mg cm^{-2}
Electrolyte	Weight	1.83 g Ah^{-1}
Package foil	Areal weight	23.1 mg cm^{-2} (double layer)
Pouch cell	Discharging energy	262.4 mWh cm^{-2}
	Total weight	716.0 mg cm^{-2}
	Gravimetric energy density	366.5 Wh Kg^{-1}

References

1. Cao J, Qian G, Lu X, Lu X. Advanced Composite Lithium Metal Anodes with 3D Frameworks: Preloading Strategies, Interfacial Optimization, and Perspectives. *Small* **19**, 2205653 (2023).
2. Zu C-X, Li H. Thermodynamic analysis on energy densities of batteries. *Energy Environ Sci* **4**, 2614-2624 (2011).
3. Wu J, *et al.* Composite Lithium Metal Anodes with Lithiophilic and Low-Tortuosity Scaffold Enabling Ultrahigh Currents and Capacities in Carbonate Electrolytes. *Adv Funct Mater* **31**, 2009961 (2021).
4. Wu J, *et al.* Polycationic polymer layer for air-stable and dendrite-free Li metal anodes in carbonate electrolytes. *Adv Mater* **33**, 2007428 (2021).
5. Liu P, *et al.* LiBr–LiF-Rich Solid–Electrolyte Interface Layer on Lithiophilic 3D Framework for Enhanced Lithium Metal Anode. *Small Struct*, 2200010 (2022).
6. Li X, *et al.* Thickness-controllable Li–Zn composite anode for high-energy and low-N/P ratio lithium metal batteries. *J Mater Chem A* **10**, 11246-11253 (2022).
7. Zhou Y, *et al.* A novel dual-protection interface based on gallium-lithium alloy enables dendrite-free lithium metal anodes. *Energy Storage Mater* **39**, 403-411 (2021).
8. Zhang Y, *et al.* Enabling 420 Wh kg^{-1} Stable Lithium Metal Pouch Cells by Lanthanum Doping. *Adv Mater* **35**, 2211032 (2023).
9. Wang A, *et al.* Stable all-solid-state lithium metal batteries enabled by ultrathin LiF/Li3Sb hybrid interface layer. *Energy Storage Mater* **49**, 246-254 (2022).
10. Pathak R, *et al.* Fluorinated hybrid solid-electrolyte-interphase for dendrite-free lithium deposition. *Nat Commun* **11**, 1-10 (2020).
11. Li S, *et al.* A robust all-organic protective layer towards ultrahigh-rate and large-capacity Li metal anodes. *Nat Nanotechnol* **17**, 613-621 (2022).
12. Luo C, *et al.* Roll-To-Roll Fabrication of Zero-Volume-Expansion Lithium-Composite Anodes to Realize High-Energy-Density Flexible and Stable Lithium-Metal Batteries. *Adv Mater* **34**, 2205677 (2022).
13. Zhang G, *et al.* A 10-μm Ultrathin Lithium Metal Composite Anodes with Superior

Electrochemical Kinetics and Cycling Stability. *Energy Environ Mater* **6**, e12598 (2023).

14. Chen H*, et al.* Free-standing ultrathin lithium metal-graphene oxide host foils with controllable thickness for lithium batteries. *Nat Energy* **6**, 790-798 (2021).
