# Supplementary material for: Hierarchical Li electrochemistry using alloy-type anode for high-energy-density Li metal batteries
Source: Nat Commun. 2024 Feb 14;15:1354. doi: 10.1038/s41467-024-45613-4 (PMC10867008; doi:10.1038/s41467-024-45613-4)
Supplement: Supplementary file 3 — Description of Additional Supplementary Files [file 41467_2024_45613_MOESM3_ESM.pdf]

Description of Additional Supplementary Files

Supplementary Data 1: Atomic coordinates of one Li^+ absorbed at the LiZn(110) surface.

Supplementary Data 2: Atomic coordinates of one Li^+ absorbed at the Li(100) surface.
